# Fpa (YlaN) is an iron(II) binding protein that functions to relieve Fur-mediated repression of gene expression in *Staphylococcus aureus*

**DOI:** 10.1128/mbio.02310-24

**Published:** 2024-10-23

**Authors:** Jeffrey M. Boyd, Kylie Ryan Kaler, Karla Esquilín-Lebrón, Ashley Pall, Courtney J. Campbell, Mary E. Foley, Gustavo Rios-Delgado, Emilee M. Mustor, Timothy G. Stephens, Hannah Bovermann, Todd M. Greco, Ileana M. Cristea, Valerie J. Carabetta, William N. Beavers, Debashish Bhattacharya, Eric P. Skaar, Lindsey N. Shaw, Timothy L. Stemmler

**Affiliations:** 1 Department of Biochemistry and Microbiology, Rutgers, the State University of New Jersey, New Brunswick, New Jersey, USA; 2 Department of Pharmaceutical Sciences, Wayne State University, Detroit, Michigan, USA; 3 Department of Molecular Biosciences, University of South Florida, Tampa, Florida, USA; 4 Department of Molecular Biology, Princeton University, Princeton, New Jersey, USA; 5 Department of Biomedical Sciences, Cooper Medical School of Rowan University, Camden, New Jersey, USA; 6 Department of Pathology, Microbiology, and Immunology, Vanderbilt University Medical Center, Nashville, Tennessee, USA; Baylor College of Medicine, Houston, Texas, USA

**Keywords:** iron, sulfur, Fur, *Staphylococcus aureus*, YlaN, Fpa

## Abstract

Iron (Fe) is a trace nutrient required by nearly all organisms. As a result of the demand for Fe and the toxicity of non-chelated cytosolic ionic Fe, regulatory systems have evolved to tightly balance Fe acquisition and usage while limiting overload. In most bacteria, including the mammalian pathogen *Staphylococcus aureus*, the ferric uptake regulator (Fur) is the primary transcriptional regulator controlling the transcription of genes that code for Fe uptake and utilization proteins. Fpa (formerly YlaN) was demonstrated to be essential in *Bacillus subtilis* unless excess Fe is added to the growth medium, suggesting a role in Fe homeostasis. Here, we demonstrate that Fpa is essential in *S. aureus* upon Fe deprivation. Null *fur* alleles bypassed the essentiality of Fpa. The absence of Fpa abolished the derepression of Fur-regulated genes during Fe limitation. Bioinformatic analyses suggest that *fpa* was recruited to Gram-positive bacteria and, once acquired, was maintained in the genome as it co-evolved with Fur. Consistent with a role for Fpa in alleviating Fur-dependent repression, Fpa and Fur interacted *in vivo*, and Fpa decreased the DNA-binding ability of Fur *in vitro*. Fpa bound Fe(II) *in vitro* using oxygen or nitrogen ligands with an association constant that is consistent with a physiological role in Fe homeostasis. These findings have led to a model wherein Fpa is an Fe(II) binding protein that influences Fur-dependent regulation through direct interaction.

**IMPORTANCE:**

Iron (Fe) is an essential nutrient for nearly all organisms. If Fe homeostasis is not maintained, Fe may accumulate in the cytosol, which can be toxic. Questions remain about how cells efficiently balance Fe uptake and usage to prevent overload. Iron uptake and proper metalation of proteins are essential processes in the mammalian bacterial pathogen *Staphylococcus aureus*. Understanding the gene products involved in the genetic regulation of Fe uptake and usage and the physiological adaptations that *S. aureus* uses to survive in Fe-depleted conditions provides insight into pathogenesis. Herein, we demonstrate that the DNA-binding activity of the ferric uptake regulator transcriptional repressor is alleviated under Fe limitation, but uniquely, in *S. aureus*, alleviation requires the presence of Fpa.

## INTRODUCTION

Iron (Fe) is an essential nutrient for nearly all organisms. Cytosolic Fe overload can cause toxicity through Fenton chemistry and/or the mismetalation of proteins (1). Because of this paradox, organisms must properly balance Fe uptake with Fe usage to ensure fitness and survival. *Staphylococcus aureus* requires Fe to proliferate and infect host tissues (2). The *S. aureus* genome codes for numerous Fe acquisition systems, including the synthesis of two siderophores and one metallophore (3, 4). *In toto*, thus far, 2% of protein-coding open reading frames are utilized for Fe uptake (3, 5). Once acquired, Fe is used to build iron-sulfur clusters (FeS) and heme and metalate proteins. The synthesis of FeS clusters is essential (6).

The ferric uptake regulator (Fur) is a master regulator of Fe uptake in bacteria (7). Few studies have examined the function of Fur in Gram-positive bacteria including phylum Firmicutes, in which *S. aureus* and *Bacillus subtilis* are members (8). The accepted model for Fur regulation is that holo-Fur acts as a transcriptional repressor or activator (9). Fur can bind various divalent metals *in vitro*, but Fe(II) is generally considered the co-repressor *in vivo*, although this has never been demonstrated (10, 11). It was recently demonstrated that *Escherichia coli* Fur binds a [2Fe-2S] cluster via four conserved C-terminal cysteine residues (12, 13). Fur had a higher affinity for DNA when ligating an FeS cluster, and a Fur variant that is incapable of binding a FeS cluster had a lower affinity for DNA *in vitro* and did not function *in vivo*, suggesting that the presence of the FeS cluster may provide a means of sensing intracellular Fe availability (14). Fur directly binds to operators (Fur boxes) and represses the transcription of genes that code for proteins involved in Fe uptake during Fe-replete conditions. When the cellular concentration of Fe is low, Fur is demetalated, and repression is alleviated. Fur often represses the expression of a small non-coding RNA (sRNA; *isrR* in *S. aureus*), which helps to optimize Fe usage by altering gene expression during Fe limitation (15–17).

The *ylaN* gene (renamed Fpa for Fur protein antagonist) is essential in *B. subtilis*, and the essentiality can be bypassed by growth with excess Fe salts (18). Decreased *fpa* expression results in phenotypes that mimic the decreased expression of *sufCDSUB*, which codes the essential FeS cluster synthesis system. These findings led us to hypothesize that Fpa functions in Fe homeostasis. In this study, we created a Δ*fpa* mutant in *S. aureus* and demonstrated that Fpa is essential when cells are cultured in low Fe conditions. The growth defects of the Δ*fpa* mutant were relieved by a null mutation in *fur*. We demonstrate that Fpa binds to Fur *in vivo*, and the interaction between Fpa and Fur decreases the DNA-binding activity of Fur *in vitro*. Therefore, Fpa functions to alleviate Fur repression during Fe limitation. Our findings support a model wherein Fpa is an Fe(II) binding protein that alters the affinity of Fur for DNA through direct interaction.

## RESULTS

### An *S. aureus* Δ*fpa* mutant is sensitive to iron chelators

**Fig 1:**
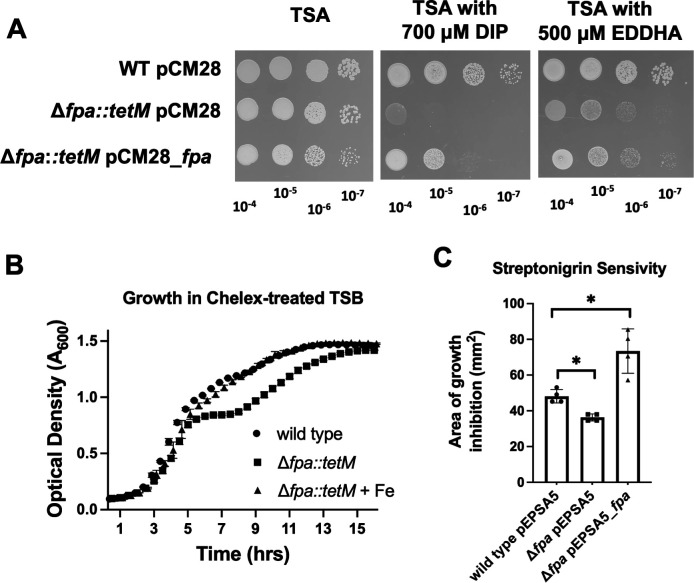
A role for Fpa in Fe ion homeostasis. (A) Growth of the WT (JMB1100) and Δ*fpa::tetM* (JMB8689) with pCM28 or pCM28_*fpa* on TSA-Cm with or without 700 µM DIP or 500 µM EDDHA. Overnight cultures were grown in TSB, serially diluted, and spot plated. Plates were incubated for 18 hours before visualization. Pictures of a representative experiment are displayed. (B) Growth of the WT and Δ*fpa::tetM* strains in Chelex-treated TSB medium supplemented with trace metals lacking Fe supplemented with and without 40 µM Fe(II). (C) Streptonigrin sensitivity was monitored using TSB-Cm top-agar overlays enclosing the WT or Δ*fpa::tetM* strains containing either pEPSA5 or pEPSA5_*fpa*. Five microliter of 2.5 mg mL^−1^ streptonigrin was spotted upon the overlays, and the area of growth inhibition was measured after 18 hours of growth. The data in panels B and C represent the average of three biological replicates with SDs displayed. Student’s *t*-tests were performed on the data in panel C, and * indicates *P* < 0.05.

We created a Δ*fpa::tetM* mutant in the community-acquired methicillin-resistant *S. aureus* isolate USA300_LAC (wild type (WT)). The growth of the Δ*fpa::tetM* strain was indistinguishable from that of WT in tryptic soy broth (TSB) medium (Fig. S1). We also generated an *fpa::Tn* strain in LAC that also grew similar to the WT in TSB. The metal ion chelators 2,2’-dipyridyl (DIP) and ethylenediamine-*N*,*N*’-bis(2-hydroxyphenylacetic acid) (EDDHA) have high affinities for Fe ions (19, 20). DIP can cross the cell membrane, whereas the larger EDDHA likely does not (21, 22). We spot-plated the WT strain with pCM28 (empty vector) or the Δ*fpa::tetM* strain containing either pCM28 or pCM28_*fpa* on tryptic soy agar (TSA) media with or without DIP or EDDHA. The Δ*fpa::tetM* strain had a growth defect on both metal starved media, and the phenotypes could be genetically complemented; however, the strain with pCM28_*fpa* had a slight growth defect compared to the WT (Fig. 1A). The fact that the Δ*fpa::tetM* strain has a growth defect with EDDHA suggests that it is defective in acquiring Fe from the environment. The finding that the Δ*fpa::tetM* strain has a growth defect with DIP suggests that the Δ*fpa::tetM* strain is defective in growth when there is competition for intracellular divalent metal ions.

To examine whether the chelator-dependent growth defects are the result of decreased Fe ion availability, we treated TSB medium with Chelex-100 and then added trace metals except Fe to the medium. The Δ*fpa::tetM* strain had a growth defect in this medium, and the growth defect was alleviated by adding Fe salts (Fig. 1B). The Fe-dependent growth defect was apparent after approximately 5 hours of growth when glucose fermentation slowed and respiratory growth, which requires more Fe-dependent enzymes, was initiated (6).

When combined with intracellular non-chelated (also called unbound or free) Fe ions and electrons, the antibiotic streptonigrin causes double-stranded DNA breaks (23). Strains hypo- or hyper-active for Fe uptake are more resistant and sensitive to streptonigrin, respectively (24). We created TSA overlays containing the WT strain with pEPSA5 (empty vector) or the Δ*fpa::tetM* strain with pEPSA5 or pEPSA5_*fpa*. The pEPSA5_*fpa* vector placed *fpa* under the transcriptional control of P*_xylRO_,* allowing for xylose induction of *fpa* expression. We subsequently spotted streptonigrin and measured the zone of growth inhibition. The Δ*fpa::tetM* strain was more resistant than the WT to growth in the presence of streptonigrin, suggesting that this strain had decreased streptonigrin-accessible cytosolic Fe (Fig. 1C). Over-production of *fpa* made cells more sensitive to streptonigrin, suggesting that overproducing Fpa increased the levels of streptonigrin-accessible Fe. The data support a model wherein a Δ*fpa* mutant has decreased Fe uptake and/or a decreased pool of non-chelated Fe ions, and over-expression of *fpa* increases the pool of cytosolic non-chelated Fe ions.

### Null mutations in *fur* suppress the growth defect of a Δ*fpa* mutant in Fe-limiting conditions

We conducted a suppressor screen to investigate *fpa* essentiality under low Fe growth conditions. We individually plated 10 cultures of the Δ*fpa::tetM* strain on TSA medium containing 700 µM DIP. We mapped the second-site mutations in the colonies that arose, and nine strains contained a single nucleotide polymorphism in the gene that codes for the Fur, resulting in the following variants: V29F (isolated twice), C103Y (isolated twice), E52stop, T62M, R61I, and R23H. One mutant had a stop codon introduced at the 11th codon (E11stop), which we decided to use as a representative allele and, henceforth, is referred to as fur*. The 10th strain had a guanine to cytosine transversion mutation in the 5′ untranslated region 13 base pairs upstream of the translation start codon of *fur*.

We compared the sequences of the *S. aureus* and *B. subtilis* Fur with sequences of Fur that have been biochemically and structurally characterized (Fig. S2). Two of the variants, R61I and R23H, had changes in residues demonstrated to be involved in DNA binding in the *Magnetospirillum gryphiswaldense* Fur (25). Two variants (V29F and T62M) contained changes in amino acids adjacent to the described DNA-binding residues. One variant, C103Y, alters one of the four Cys residues that ligate Zn(II) in the *Helicobacter pylori* (26), *Campylobacter jejuni* (27), and *Francisella tularensis* (28) Fur X-ray structures.

**Fig 2:**
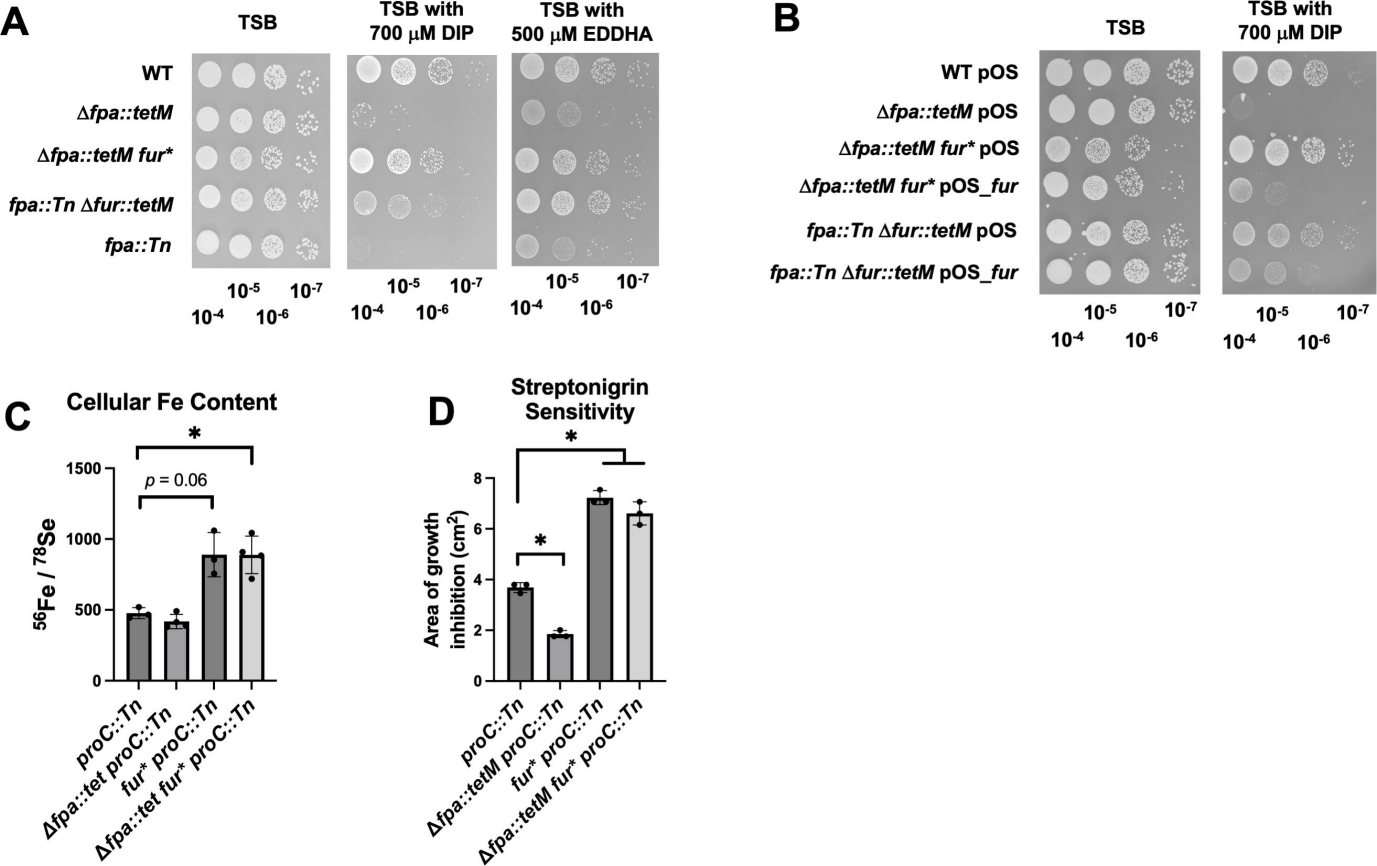
Null *fur* mutation bypasses the essentiality of Fpa in Fe-deplete conditions. (A) Overnight cultures of the WT (JMB1100), Δ*fpa::tetM* (JMB8689), Δfpa::tetM fur* (JMB10678), *fpa::Tn fur::tetM* (JMB10641), and *fpa::Tn* (JMB8428) strains were serially diluted and spot plated on TSB media with or without 700 µM DIP or EDDHA. (B) Overnight cultures of the WT, Δ*fpa::tetM*, Δfpa::tetM fur*, and *fpa::Tn fur::tetM* strains containing either pOS or pOS_*fur* were serially diluted and spot plated on TSB-Cm media with or without 700 µM DIP. (C) The total (bound and unbound) ^56^Fe and ^78^Se loads were quantified in whole cells using inductively coupled plasma mass spectrometry (ICPMS) after culture in TSB medium. The ratio of ^56^Fe/^78^Se is displayed for the *proC::Tn* (JMB10675), Δ*fpa::tetM proC::Tn* (JMB10677), fur* proC::Tn (JMB10676), and Δfpa::tetM fur* proC::Tn (JMB10678) strains. (D) Streptonigrin sensitivity was monitored using top-agar TSA overlays enclosing the *proC::Tn*, Δ*fpa::tetM proC::Tn*, fur* proC::Tn, and Δfpa::tetM fur* proC::Tn strains. Five microliter of 2.5 mg mL^−1^ streptonigrin was spotted upon the overlays, and the area of growth inhibition was measured after 18 hours of growth. Pictures of representative experiments are displayed in panels A and B after 18 hours of growth. The data in panels C and D represent the average of three biological replicates with SDs displayed. Student’s *t*-tests were performed on the data in panels C and D. * indicates *P* < 0.05.

We linked a transposon (Tn; SAUSA300_1452; *proC::Tn*) to the fur* allele and then transduced the fur* allele into the WT and Δ*fpa::tetM* strains by selecting for *proC::Tn*. Some transductants had the fur* allele, while others retained the WT copy of *fur*. All transductants with fur* suppressed the growth defect of Δ*fpa::tetM* strain on TSA medium with DIP, whereas those containing the WT *fur* allele did not (Fig. 2A). Two results demonstrate that the fur* allele is null and recessive. First, we found that a Δ*fur::tetM* allele suppresses the DIP and EDDHA sensitivity phenotypes of a *fpa::Tn* mutant (Fig. 2A). Second, we were able to genetically complement the fur* and Δ*fur::tetM* alleles with wild-type *fur* (Fig. 2B).

We quantified the total Fe load (bound by macromolecules and free) in the parent, Δ*fpa::tetM* mutant, and isogenic fur* strains after growth in TSB. There was a significant increase in the abundance of Fe relative to the abundances of Se or *P* in the fur* mutant strains compared to the parent and Δ*fpa::tetM* strains (Fig. 2C; Fig. S3). The fur* allele also increased the streptonigrin sensitivity of the Δ*fpa::tetM* mutant, consistent with the null *fur* allele increasing the size of the non-chelated Fe pool (Fig. 2D). Introduction of a *fhuC::Tn* mutation, which inactivates the three siderophore-dependent Fe uptake systems (29, 30), into the Δ*fur::tetM* strain decreased the sensitivity to streptonigrin, demonstrating that the increased streptonigrin sensitivity of strains with a null *fur* mutation is the result of increased Fe uptake (Fig. S4). These data demonstrate that the fur* allele increased the total Fe load and the amount of intracellular non-bound Fe.

**Fig 3:**
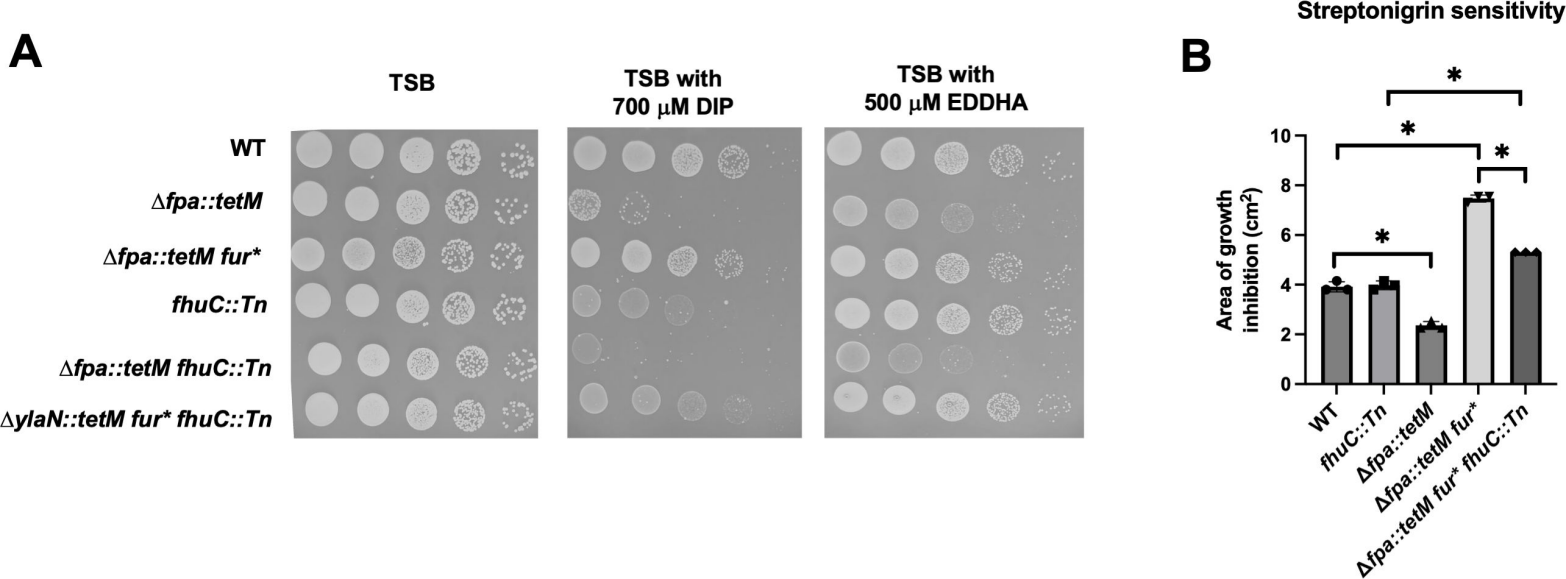
Inhibiting high-affinity Fe uptake decreases the ability of the fur* allele to suppress the growth defect of the Δ*fpa::tetM* mutant during Fe starvation. (A) Growth of the WT (JMB1100), Δ*fpa::tetM* (JMB8689), Δfpa::tetM fur* (JMB10678), *fhuC::Tn* (JMB7525)*,* Δ*fpa::tetM fhuC::Tn* (JMB10721), and Δfpa::tetM fur* fhuC::Tn (JMB10722) strains with or without 700 µM DIP or 500 µM EDDHA. Overnight cultures were grown in TSB, serially diluted, and spot plated. Pictures of a representative experiment are displayed. (B) Streptonigrin sensitivity was monitored using top-agar TSA overlays enclosing the WT, *fhuC::Tn,*Δfpa::tetM fur* (JMB10638), and Δfpa::tetM fur* fhuC::Tn (JMB10722) strains. Five microliter of 2.5 mg mL^−1^ streptonigrin was spotted upon the overlays, and the area of growth inhibition was measured after 18 hours of growth. The bars represent the average of three biological replicates with SDs displayed. Student’s *t*-tests were performed on the data in panels C and D. * indicates *P* < 0.05.

We examined whether the fur* allele was alleviating the DIP phenotype of the Δ*fpa::tetM* strain by promoting Fe uptake. The *fhuC::Tn* strain had a growth defect on a solid TSB medium containing DIP, but the phenotype was less severe than that of the Δ*fpa::tetM* strain (Fig. 3A). The Δfpa::tetM fur* fhuC::Tn strain phenocopied the growth defect of the *fhuC::Tn* strain. The Δ*fpa::tetM* strain had a slow growth phenotype on solid TSB with EDDHA, whereas the *fhuC::Tn* and Δfpa::tetM fur* fhuC::Tn strains did not. The Δfpa::tetM fur* fhuC::Tn strain had decreased streptonigrin sensitivity compared to the Δfpa::tetM fur* strain; however, the Δfpa::tetM fur* fhuC::Tn strain was more sensitive to streptonigrin than the *fhuC::Tn* (Fig. 3B). These data suggest that the fur* mutation, at least in part, suppresses the lethality of the Δ*fpa::tetM* mutant under Fe limitation by increasing expression of Fe uptake systems.

### Fpa alleviates Fur-dependent repression of transcription

**Fig 4:**
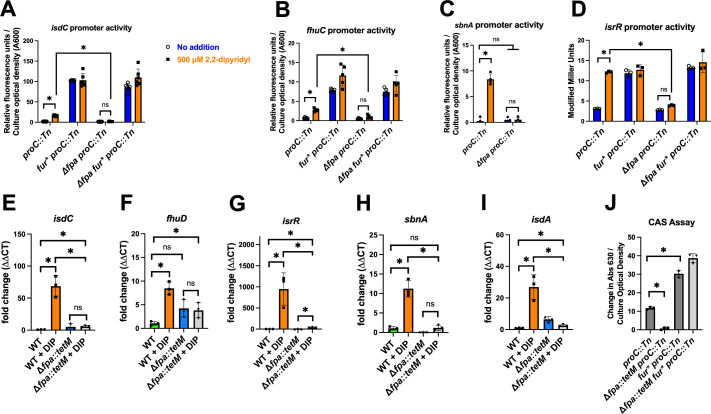
The presence of Fpa promotes Fur-dependent derepression in Fe-deplete conditions. (A–D) The transcriptional activities of the *isdC*, *fhuC*, *isrR*, and *sbnA* promoters were measured in the parent (*proC::Tn*; JMB10675), Δ*fpa::tetM proC::Tn* (JMB10677), fur* proC::Tn (JMB10676), and Δfpa::tetM fur* proC::Tn (JMB10678) strains after culture in TSB-Cm media with 500 µM DIP (orange bars) or vehicle control (blue bars). We were unable to mobilize the *sbnA* transcriptional reporter into *fur* mutant strains. The data in panels A, B, and C represent the average of biological quintets, and SDs are displayed. The data in panel D represented biological triplicates. (E–I) Abundances of RNAs expressed from select Fur-regulated genes in the WT and Δ*fpa::tetM* strains were determined using quantitative PCR after culture in TSB media with and without 500 µM DIP. The data displayed represent the average of biological triplicates, and SDs are displayed. (J) The ability of cell-free culture spent medium to compete with chrome azurol S (CAS) for Fe was measured spectrophotometrically. Cell-free spent culture medium was harvested from the *proC::Tn* (JMB10675), Δ*fpa::tetM proC::Tn* (JMB10677), fur* proC::Tn (JMB10676), and Δfpa::tetM fur* proC::Tn (JMB10678) strains after culture in Chelex-treated TSB. The data displayed represent the average of biological triplicates with SDs shown. Student’s *t*-tests were performed on the data, and * indicates *P* < 0.05.

We tested the hypothesis that Fpa alleviates Fur-dependent repression of transcription under Fe limitation. The *fhuC*, *isdC*, *isrR*, and *sbnA* genes have Fur-box consensus sequences in their promoters, suggesting direct Fur regulation (31). Consistent with this, *fhuC*, *isdC*, and *isrR* transcriptional reporters had increased reporter gene expression in the fur* strains compared to the parent strain (Fig. 4A through D). We were unable to transduce the *sbnA* reporter into *fur* mutant strains. All four promoters had increased activity in the parent strain upon growth with DIP (Fig. 4A through D). In contrast, the promoters did not significantly respond to DIP in the Δ*fpa::tetM* mutant. The transcriptional activities of the *fhuC*, *isdC*, and *isrR* promoters were indistinguishable in the Δfpa::tetM fur* double mutant strain and fur* strain. The results of these epistasis experiments are consistent with a model wherein the effects of Fpa on these promoters during Fe-limiting growth require Fur.

We quantified RNA transcripts corresponding to *isdC*, *fhuD*, *isrR*, *sbnA*, and *isdA* in the WT and Δ*fpa::tetM* strains after growth with and without DIP. All transcripts increased in WT upon DIP challenge; however, none of these transcripts significantly accumulated in the Δ*fpa::tetM* strain in response to DIP (Fig. 4E through I). Consistent with *isdC*, *fhuD*, *isrR*, *sbnA*, and *isdA* being under Fur transcriptional control, all transcripts were significantly increased in a Δ*fur::tetM* mutant as compared to WT (Fig. S5).

We monitored siderophore production using chrome azurol S (CAS). Fe-bound CAS is blue in color. Siderophores compete with CAS for the Fe resulting in apo-CAS, which is orange. We cultured the parent (*proC::Tn*), Δ*fpa::tetM*, fur*, and Δfpa::tetM fur* strains in TSB before measuring CAS competition using spent media. The spent media from the Δ*fpa::tetM* strain did not alter the absorbance of the CAS-Fe complex, whereas the spent media from the parent strain did. Spent media from the fur* and Δfpa::tetM fur* strains were significantly better able to compete for Fe than the parent, and the two strains containing fur* phenocopied one another (Fig. 4J). These data are consistent with the transcriptional reporter data reported in Fig. 4 and strengthened our working model wherein Fpa is required to express the high-affinity (siderophore) Fe uptake systems when Fur is present.

### Fpa and Fur likely co-evolved

**Fig 5:**
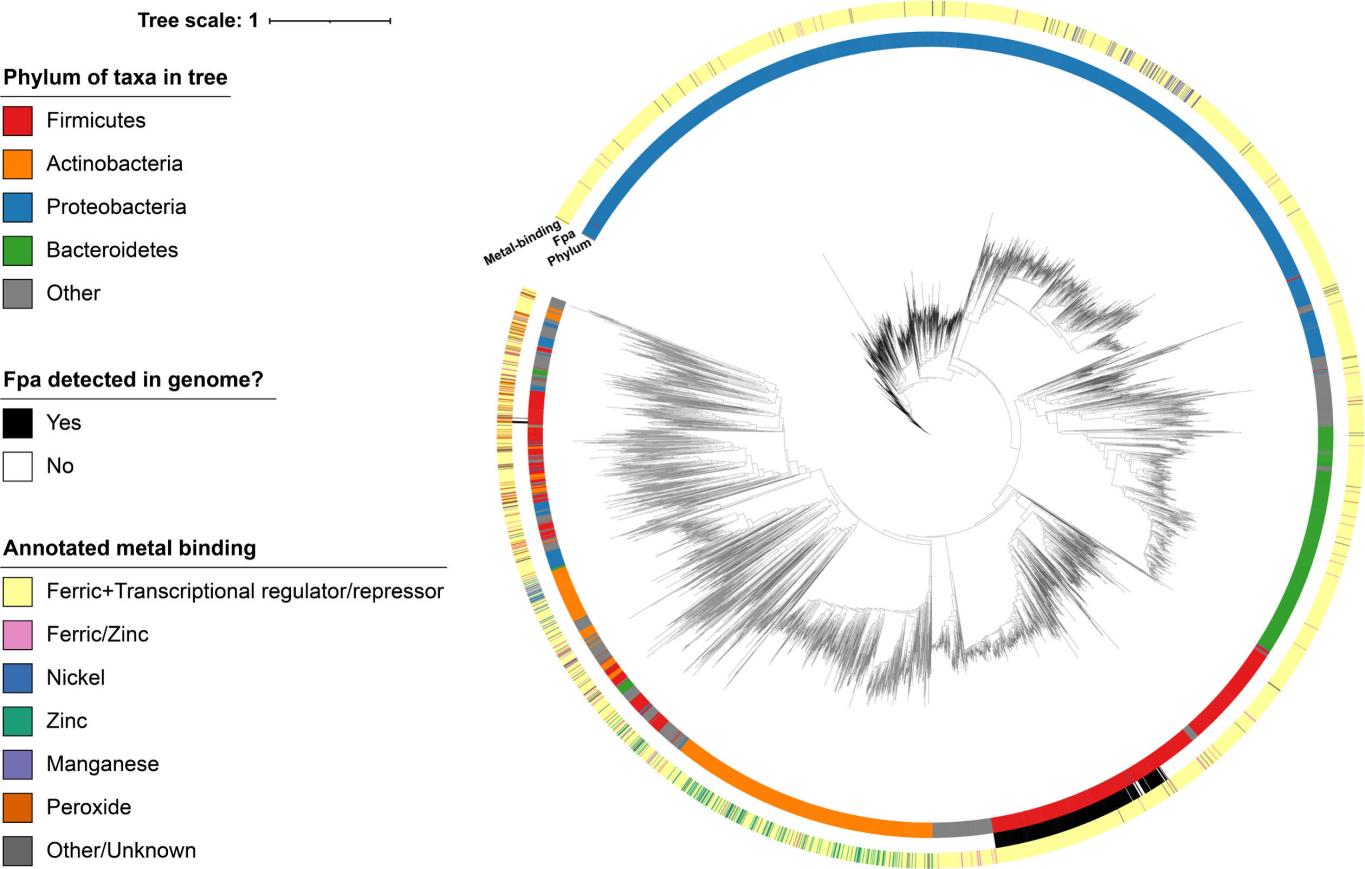
Phylogeny of representative bacterial Fur sequences. Tree (midpoint rooted; built using the fastme program) of Fur protein sequences retrieved from bacterial genomes available through NCBI. Given a large number of bacterial sequences currently available through NCBI, down sampling of the retrieved Fur sequences to a genera level (i.e., only one genome per bacterial genus was considered), from just high-quality representative or reference bacterial genomes, was performed to constrain the number of sequences in the tree. For each sequence in the tree, the colors in the surrounding rings represent the phylum of the genome from which the Fur sequence was derived (inner ring), the presence or absence of F in the genome from which the Fur sequence was derived (middle ring), and annotated metal-binding affinity on NCBI of each Fur sequence (outer ring). A legend showing the colors used in the surrounding rings is shown on the left side of the image and the branch length scale.

We compared the conservation of *fpa* and *fur* in bacterial genomes. The presence of *fpa* is restricted to Firmicutes, forming a clear monophyletic clade (Fig. 5). The Fur sequences from Fpa-positive genomes are paraphyletic, with the sequences from Fpa-negative genomes having diverged early in this group. This suggests that Fpa was lost from these lineages during the early stages of its evolution. There are only six sequences in the tree (all Firmicutes) that are from Fpa-positive genomes but are not part of the monophyletic clade of Firmicutes Fur sequences. These sequences are positioned with non-Fur sequences in the tree (e.g., Zur and PerR) and are likely to be PerR. They were recruited into the tree during the DIAMOND (sequence similarity-based) search and thus support the singular origin of Fpa in Firmicutes.

Comparison of the Firmicutes Fur and Fpa phylogenies (Fig. S6 and S7) demonstrates that while there is significant discordance between both trees, generally, many of the closely related groups of sequences are present in both trees (represented by the colored lines), and the major differences arise from the early diverging nodes which are weakly supported (i.e., have bootstrap support <95%). This result suggests that Fur and Fpa have been co-evolving, although, given the lack of phylogenetic signal for the early diverging nodes in both protein trees, this hypothesis is currently provisional.

### Fpa and Fur interact *in vivo*

The findings that Fpa alters the ability of Fur to respond to Fe limitation, and Fpa and Fur likely co-evolved in the Firmicutes led to the hypothesis that these two proteins physically interact. Supporting this hypothesis, immunoaffinity purification of Fur from *B. subtilis* resulted in an enrichment of Fpa (32). We conducted the reciprocal experiment and performed an immunoaffinity purification of Fpa in *B. subtilis*. We conducted these experiments in *B. subtilis* because the immunopurification protocol conditions are optimized (33) and for safety concerns. *B. subtilis*, like *S. aureus*, is a member of the phylum Firmicutes.

We created a strain encoding an N-terminally fused *yfp_fpa* construct expressed from the *amyE* locus, which was under the transcriptional control of an isopropyl β-D-1-thiogalactopyranoside (IPTG)-inducible promoter. To confirm the functionality of YFP-Fpa, we took advantage of the conditional lethality of Δ*fpa* mutants (18). In the presence of excess Fe, a *fpa* deletion was able to be constructed, but growth was completely dependent upon Fe supplementation (Fig. S8). In the presence of YFP-Fpa, deletion of *fpa* was not dependent upon the presence of excess Fe, demonstrating the functionality of the construct.

YFP-Fpa was immunopurified from both logarithmic and stationary phase cells, and co-isolated proteins were identified by mass spectrometry. To control for non-specific interactions, we immunopurified Gfp from an isogenic strain expressing an inducible *gfp* construct from the *amyE* locus. Fur was the most abundant protein co-isolated with YFP-Fpa with high specificity (>40-fold spectral count enrichment vs the GFP control) in both exponential and stationary phases (Table S1), supporting the hypothesis that Fpa interacts with Fur.

We examined whether Fur and Fpa interact in *S. aureus* cells. For these experiments, we used a two-plasmid split luciferase system. The plasmids individually code for the C- or N-terminal portions of luciferase. We cloned *fur* or *fpa* into each plasmid to be expressed in frame with either the small (SmBIT) or large (LgBIT) portion of luciferase. If Fur and Fpa interact, the two portions of luciferase are brought into contact, resulting in a functional luciferase complex.

**Fig 6:**
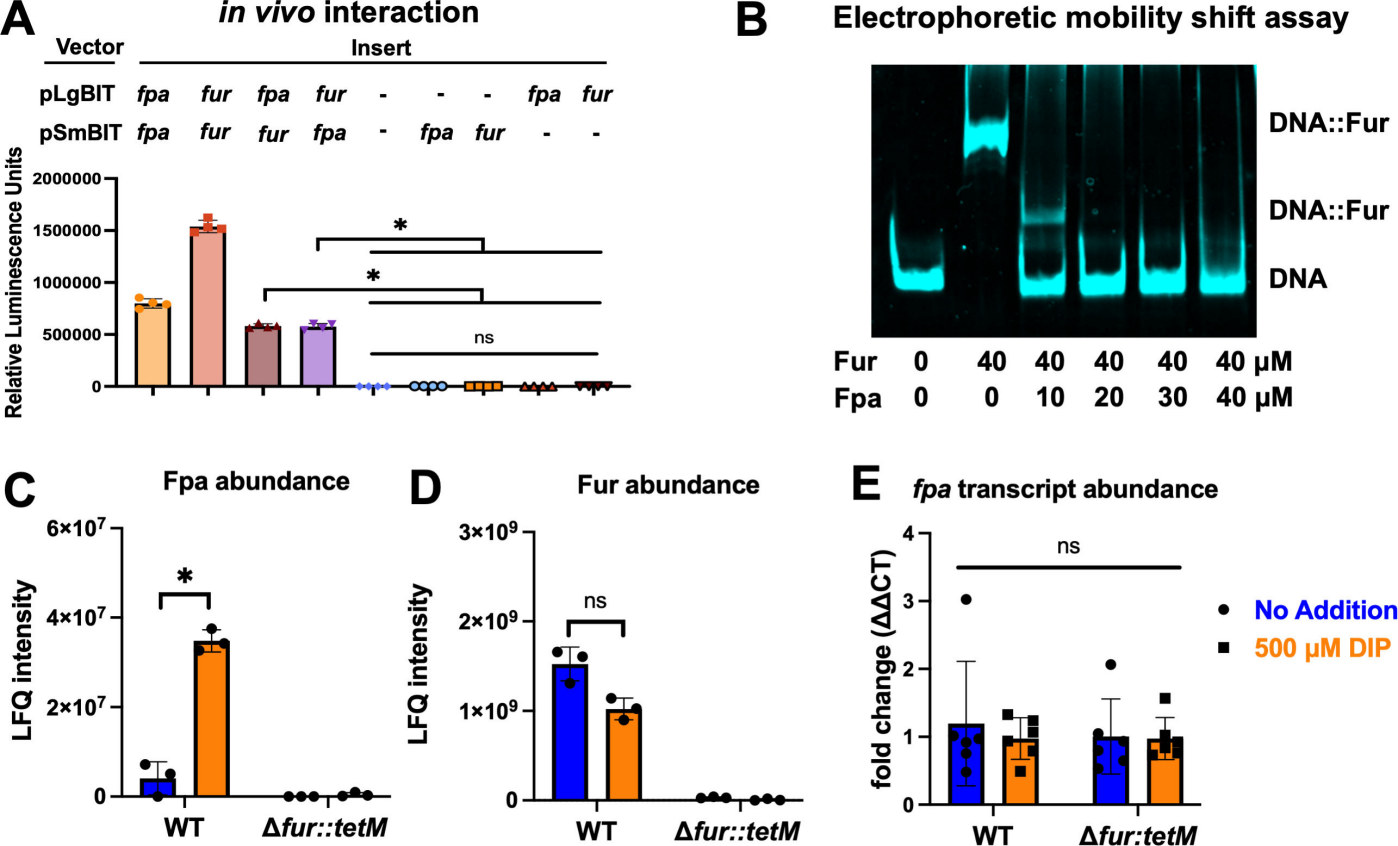
Fur and Fpa interactions. (A) Fpa-Fur interaction was assessed in an *S. aureus* Δfpa::tetM fur* (null mutation; JMB10678) double mutant using a two plasmid (pLgBIT and pSmBIT) split luciferase system after growth in TSB. Luminescence was used as a measure of protein-protein interaction. The data displayed represent the average luminescence of four biological replicates with SDs displayed. (B) Electrophoretic mobility shift assay (EMSA) demonstrates Fpa inhibition of Fur binding to Fur box consensus DNA. Fpa was titrated (0–40 µM) into an EMSA binding reaction containing Fur (40 µM) and *isdC* operator DNA. (C) The label-free quantification (LFQ) intensity of Fpa (UniProt ID: Q2FHW8) was quantified in cell-free lysates generated from the WT (JMB1100) and Δ*fur::tetM* (JMB1432) strains after culture in TSB media with (orange bars) and without (blue bars) 500 µM DIP. The raw proteomics data can be found in Table S2. (D) The LFQ intensity of Fur (UniProt ID: A0A0H2XGT2) was determined in cell-free lysates generated from the WT and Δ*fur::tetM* strains after culture in TSB media with (orange bars) and without (blue bars) 500 µM DIP. (E) The abundances of mRNA transcript corresponding to *fpa* were measured using quantitative PCR after culture of the WT and Δ*fur::tetM* strains in TSB media with (orange bars) and without (blue bars) 500 µM DIP. The data displayed in panels C and D are presented in Table S2 and represent the average of three biological replicates with SDs shown. The data displayed in panels A and E represent the average of four and six biological replicates, respectively, and SDs are shown. Student’s *t*-tests were performed on the data in panels A, C, D, and E, and * indicates *P* < 0.05.

We first confirmed that the chimeric Fpa and Fur proteins functioned *in vivo* using genetic complementation (Fig. S9 and S10). We then transduced the plasmids into the fur* Δ*fpa::tetM* strain. *S. aureus* containing both empty vectors or a combination of an empty vector and a construct coding for a chimeric Fur or Fpa did not produce detectable luciferase activity (Fig. 6A). However, strains producing two Fur, two Fpa, or one Fur and one Fpa, had detectable luciferase activity. These data are consistent with previous findings that Fur and Fpa form homodimers (34, 35) and support the hypothesis that Fur and Fpa interact in *S. aureus*.

### Fpa decreases the DNA-binding activity of Fur *in vitro*

We examined Fur binding to the *isdC* operator, which has two Fur boxes, using a binding electrophoretic mobility shift assay (EMSA) (36, 37). We were unable to include Fe(II) in these assays and ensure that it was not oxidized. Instead, we included Mn(II), which has been demonstrated to act as a co-repressor with Fur at high concentrations (25). Combining purified *S. aureus* Fur with the *isdC* operator DNA resulted in a retardation in DNA migration consistent with Fur binding the DNA (Fig. S11). There was a concentration-dependent increase in binding, with 40 µM Fur being required for complete binding to the DNA.

We next examined the effect of Fpa on the ability of Fur to interact with the *isdC* operator. We fixed the Fur concentration for this experiment at 0 or 40 µM and titrated in *S. aureus* Fpa. The addition of 20 µM Fpa fully reversed Fur association with DNA (Fig. 6B). These data are consistent with a model wherein Fpa decreases the ability of Fur to associate with DNA.

### Fpa abundance is altered by the presence of Fur

We conducted a proteomics study to quantify the abundances of Fur and Fpa in *S. aureus* cells. We cultured the WT and Δ*fur::tetM* strains in TSB with and without 500 µM DIP. Cell-associated solubilized proteins were isolated and subjected to mass spectrometry analysis to quantify total abundances. As expected, there was an overlap between proteins that increased or decreased in abundance in the WT treated with DIP and Δ*fur::tetM* mutant (Table S2). Unexpectedly, the label-free quantification (LFQ) intensity was approximately 380-fold higher for Fur than for Fpa in the WT strain under non-challenged growth (Fig. 6C and D). The LFQ intensity for Fpa increased when cells were cultured with DIP, and the ratio of Fur to Fpa LFQ intensity decreased to approximately 30. Peptides that mapped to Fpa were nearly undetectable in the Δ*fur::tetM* strain. There was not a significant difference in the LFQ intensity for Fur in the WT cells cultured with or without DIP (Fig. 6D).

We tested the hypothesis that *fpa* transcription is regulated by Fur and divalent metal ions. We cultured the WT and Δ*fur::tetM* strains in TSB with and without 500 µM DIP, isolated RNA, and quantified *fpa* transcripts. The transcript levels of *fpa* do not change in the Δ*fur::tetM* mutant when compared to WT or in the divalent metal ion-depleted environment induced by the addition of DIP (Fig. 6E). These findings agree with a large-scale *S. aureus* transcriptomics study that demonstrated that *fpa* transcript abundance is not significantly altered under a wide variety of growth phases, growth media, or challenges, including growth with DIP (Fig. S12). These data support the premise that Fur influences Fpa abundance independent of transcription.

### *S. aureus* Fpa binds Fe(II), Mn(II), and Zn(II) with physiological relevant affinities

**Fig 7:**
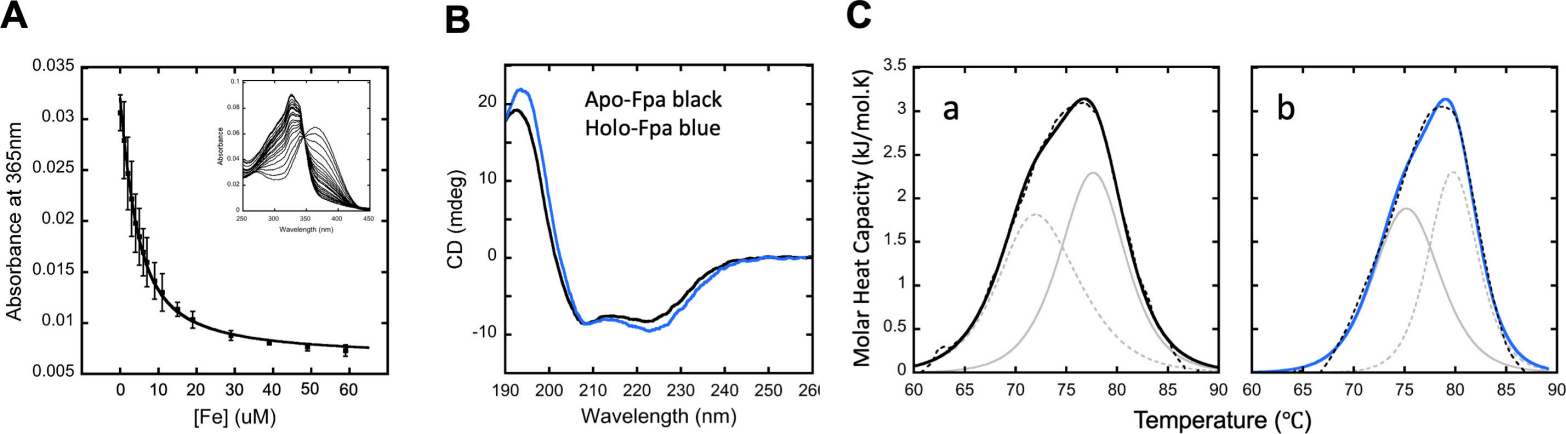
Fpa is an Fe(II) binding protein. (A) Fe-binding affinity to Fpa measured using Mag-Fura-2 within a competition-based assay. Average titration spectral points of Fe(II) ions into Fpa with overall simulation in solid line with representative titration absorption spectra inset display. Mag-fura-2 to protein ratios were varied, and 1:1 data are shown. Spectra were collected in duplicate using independent samples to ensure spectral reproducibility. (B) Circular dichroism (CD) structural results for apo and Fe(II) loaded holo-Fpa. Representative CD spectra comparing apo- (black) and holo- (blue) Fpa. (C) Differential scanning calorimetric spectra for apo- and Fe(II) loaded holo-Fpa. Overall simulation of molar heat capacity vs temperature spectra for apo-Fpa (A) and holo-Fpa (B). Overall simulations are displayed in solid lines, with individual simulated features in gray and raw data shown in a black dashed line.

We purified recombinantly produced Fpa, and we were unable to detect Fe associated with Fpa using a ferene-based assay (24). We tested the hypothesis that Fpa binds Fe(II) using a competition assay (38–40). This assay measures the Fe(II) binding affinity of a protein under heterogeneous solution conditions, which better mimics *in vivo* conditions where multiple species are present that compete for the metal ions. We measured changes in the UV-Vis signal at 365 nm from the apo-form of the chelator Mag-Fura-2, which decreases upon Fe(II) loading of the chelator. Binding analyses were performed on two independently prepared protein samples. Data were simulated to deconvolute the protein-metal binding relative to the known chelator metal binding affinity [for Mag-Fura-2, Fe(II) binding *K*_d_ = 6.5 ± 0.5 µM]. The Fe(II) binding affinity for Fpa was best fit by one independent *K*_d_ at 2.07 ± 0.46 µM (Fig. 7A).

Next, we used the anaerobic divalent metal competition assay to measure the binding affinities for Zn(II), Mg(II), and Mn(II) ions. Fpa bound Zn(II) ions, and the data were best fit by a two-site-binding model, producing the two independent *K*_d_ values of 1.8 ± 0.1 and 13.6 ± 0.1 µM (Fig. S13; Table S3). Fpa also bound Mn(II) ions, and the data were best fit by a two-site binding model, producing the two independent *K*_d_ values of 15.0 ± 0.3 and 25.6 ± 0.7 µM (Fig. S14; Table S3). The binding of Mg(II) was weak (high millimolar range) to negligible, and the binding affinity for Mg(II) was outside of the suitable detection range of the competition assay.

### Fe(II) binding to Fpa alters its secondary structure and thermal stability

We examined the impact of Fe(II) binding on the secondary structure of Fpa using circular dichroism (CD) spectroscopy. Comparison of average CD spectra of the apo- and holo-Fpa shows some change between the two protein states (Fig. 7B), with similar negative features at 207 and 223 nm; wavelengths attributed to ⍺-helical structures (41). These negative features become more prominent upon Fe(II) binding. Simulation results are shown in Table S4. These results show an overall trend of increasing helical structure coupled with Fe(II) binding. The helical content of Fe(II) loaded protein is slightly increased (from 31% in the apo state to 40% in the holo), while the relative values of the additional structural elements only slightly decrease, suggesting that upon Fe(II) binding, Fpa increases its overall helical structure. Root-mean-square deviations between theoretical and empirical data for both apo- and holo-Fpa CD spectra are consistently low, suggesting simulations accurately predict the secondary structure change for each construct.

We examined the thermal stability of apo- and Fe(II) bound Fpa using differential scanning calorimetry (DSC). The apo-protein had a two-phase thermal stability profile (Fig. 7C; Table S5). Melting temperatures for apo-Fpa were 72.0°C and 77.5°C. Fe(II) binding to Fpa increased the melting temperatures; the lower *T*_m_ was increased to 75.3°C, while the higher *T*_m_ value was increased to 79.8°C, indicating that Fe(II) binding increased the protein’s thermal stability.

### The Fpa Fe(II) binding environment consists of only oxygen and/or nitrogen ligands

We probed the structure and electronic properties of Fe(II)-loaded Fpa to determine bound-metal coordination geometry. Structural details for Fe(II) bound to Fpa were measured using Fe *k*-edge X-Ray Absorption Spectroscopy (XAS) (42). Normalized X-ray Absorption Near Edge Spectroscopy (XANES) data were used to determine the average metal oxidation state, spin state, and ligand coordination symmetry (38). The XANES edge shape (Fig. S15, left) and first inflection edge energy at around 7,123 eV (Table S8) suggest Fe-loaded Fpa contains exclusively Fe(II). The pre-edge feature (Fig. S15, right) between 7,110 and 7,115 eV represents the Fe(II) 1s->3 d electron transition. The area under the curve (Table S6) indicates that protein-bound Fe(II) is held in a highly symmetric 6-coordinate metal-ligand environment.

The strength of XAS is the high accuracy seen in the metal-ligand bond length values obtained by simulating the Extended X-Ray Absorption Fine Structure (EXAFS) portion of the XAS spectrum. In review, simulations of EXAFS data provide metal-ligand bond lengths at an accuracy of ±0.02 Å, while information regarding metal-ligand coordination numbers are obtained at an accuracy of ±1.0 and ligand identity narrowed to within one row of the periodic table (42). All Fe data were fit to a *k*-space value of 12.5 Å^−1^ to eliminate high energy noise. Calibration from Fe(II) theoretical model compounds were used for E_0_ and Sc parameters. E_0_ values for Fe-O, Fe-N, Fe-C were set at −10 eV, and a scale factor of 0.95 was used to fit the data. Figure S16 shows the raw and simulated EXAFS and Fourier Transform of the EXAFS. Best fit simulations are listed in Table S7, which fit O/N as nearest neighbor ligands, followed by C as long-range ligands. These data are consistent with a highly symmetric, octahedral Fe(II) coordination environment consisting of O/N ligands. The presence of C ligand scattering as a long-range interaction indicates Fe(II) in the sample with higher order, consistent with attachment to amino acids, as opposed to being bound adventitiously. Under these parameters, the average Debye Waller Factor and F’ values, measures of absorber-scatter bond disorder, and overall simulation convergence between theoretical and empirical data, were highly favorable.

## DISCUSSION

We initiated this study after data produced by Peters et al. suggested that Fpa had a role in Fe ion homeostasis in *B. subtilis* (18). After determining that Fpa was essential in *S. aureus* under Fe-limiting conditions, we used a non-biased genetic screen to discover that strains with mutations that inactivate *fur* bypass the need for Fpa under Fe-starvation. Fur is a nearly ubiquitous protein in bacteria that directly represses the expression of genes that function in Fe uptake, processing, and storage under Fe-replete conditions. Fur also indirectly represses the expression of genes whose gene products are involved in Fe-utilizing processes under Fe-deplete conditions (15). These findings suggested that without Fpa, Fur was repressing the transcription of genes, which became necessary upon Fe limitation. The introduction of a *fhuC::Tn* mutation, which inactivates the *S. aureus* high-affinity Fe uptake systems, partially prevented a null *fur* allele from suppressing the essentiality of Fpa under low Fe conditions. This finding suggested that proper expression of Fe uptake systems is partially responsible for the ability of the fur* alleles to bypass the need for Fpa under Fe starvation. The partial effect of the *fhuC::Tn* mutant was unsurprising since siderophore-mediated Fe uptake is not the only means for Fe acquisition in *S. aureus*.

We and others recently discovered a Fur-regulated sRNA in *S. aureus* called IsrR that decreases the expression of specific Fe-requiring proteins or processes to spare Fe ions for essential metabolic processes (15–17). These findings led to a model wherein Fpa alleviates Fur repression during Fe limitation, which results in increased Fe uptake and IsrR-dependent prioritization of cytosolic Fe usage. Consistent with this model, the Δ*fpa* mutant has decreased streptonigrin sensitivity compared to the wild type, suggesting a lower concentration of non-chelated intracellular Fe ions. The streptonigrin phenotype was nullified by the introduction of a null *fur* mutation.

The DNA-binding activity of Fur is modulated by association with divalent metal ions, and the physiological metal is thought to be Fe(II) (28). It was recently demonstrated that some Fur proteins contain C-terminal cysteine residues in a CXXC motif that can bind a 2Fe-2S cluster that bridges the dimer interface using two cysteine ligands from each monomer (13, 14). These same cysteines bind a Zn(II) in other Fur (26, 28, 43) and are not conserved in all Fur orthologs (Fig. S2), prompting questions about their role in Fur regulation. We isolated two strains with a *fur*_C103Y_ mutation, resulting in a null allele. Cysteine 103 is a predicted Zn or FeS cluster ligand. Future studies using a mutant with a more modest change, such as a Fur_C103S_ variant, will be necessary to determine if a defect in Zn or FeS cluster binding, dimer disruption, or an alternate mechanism is resulting in the inability of the Fur_C103Y_ variant to repress transcription.

We determined that *S. aureus* Fur bound the *isdC* operator DNA and complete binding required 40 µM Fur. Alternate studies have seen complete Fur DNA association with a lower concentration of Fur (37, 44). The differences could be attributed to the *S. aureus* Fur having a lower affinity for the *isdC* operator. It could also be the result of Mn(II) not acting as a co-repressor of *S. aureus* Fur. Lastly, we used SYBR green to visualize the DNA, which has a decreased sensitivity compared to assays detecting radiolabeled DNA. This resulted in more DNA being required in our assays. In the future, more sensitive techniques, such as fluorescence anisotropy, will be used to quantify the effect of Fpa, Mn(II), or alternate metals on the affinity of *S. aureus* Fur for DNA.

Until recently, as mentioned above, it was believed that the transcriptional regulation capacity of Fur was solely dictated by the presence of Fe(II). It was recently demonstrated that in some bacteria, the DNA-binding activity of Fur is also modulated by a secondary protein. In the Gram-negative bacteria *Salmonella enterica* and uropathogenic *E. coli*, the DNA-binding capability of Fur is inhibited by a direct interaction with one and two other proteins, respectively (45, 46). Similarly, data herein demonstrate that Fur and Fpa interact *in vivo*, and the presence of Fpa decreases the ability of Fur to bind DNA *in vitro*. The difference between these proteins (PtsN and YdiV) and Fpa is that Fpa was required to alleviate Fur-mediated repression in Fe-deplete conditions. All three studies demonstrate that these proteins allow for fine-tuning of Fur regulation and, in the case of PtsN and YdiV, tether other physiological processes to regulating Fe ion homeostasis through their interactions with Fur.

**Fig 8:**
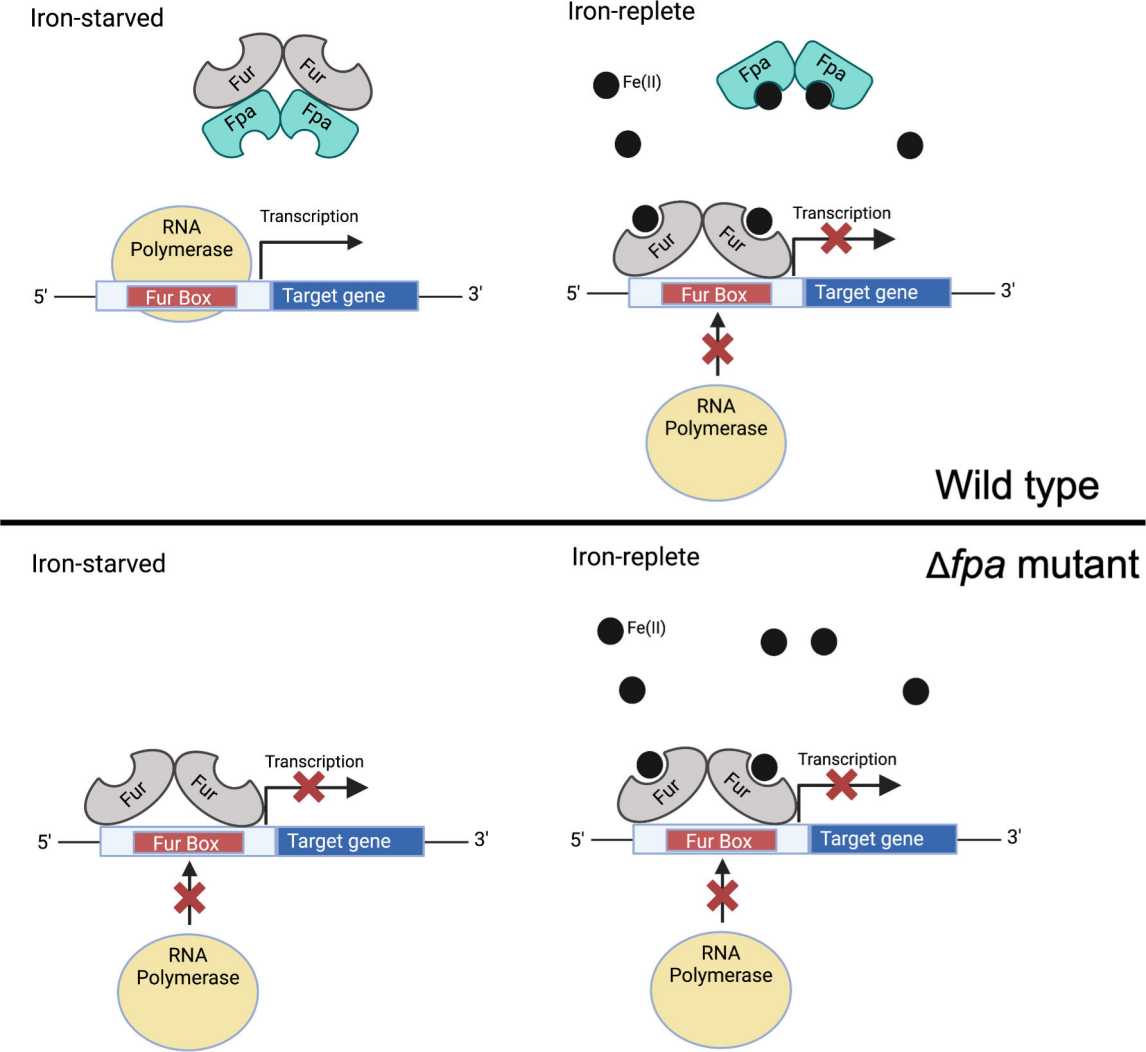
Working model for Fpa function. In our model, Fur (gray) and Fpa (cyan) bind Fe(II) transiently. They are both metalated in Fe-replete growth conditions, and Fur directly prevents transcription of genes coding for Fe acquisition machinery via binding to a consensus sequence of DNA in the operator termed a Fur box. Upon Fe limitation, Fpa forms a complex with Fur preventing Fur from binding to Fur box DNA, thus alleviating Fur repression of target genes. The oligomeric state of the heterocomplex is currently unknown.

The data presented in this study have resulted in a working model (Fig. 8) wherein during Fe-replete conditions, holo-Fur binds to operators of genes utilized in Fe uptake and represses transcription. During Fe limitation, Fpa interacts with Fur and alleviates repression. This results in the transcription of Fur-regulated Fe uptake genes and *isrR*. Increased *isrR* expression decreases the expression of genes utilized in Fe-requiring processes. In this model, Fe(II) limitation alone is not enough to relieve Fur repression, and Fpa is necessary for derepression.

For this model to be correct, the concentration of Fpa should be comparable to the concentration of Fur. We found that under Fe-replete conditions, the ratio of Fur to Fpa was approximately 600. However, upon decreased access to divalent metals imparted by co-culture with DIP, the ratio of Fur to Fpa decreased to 30. In the Δ*fur* mutant, the concentration of Fpa was nearly undetectable, demonstrating that the presence of Fur affects Fpa levels. Transcription of *fpa* was not altered in a Δ*fur* mutant or upon divalent metal chelation, and the *fpa* promoter does not contain a Fur box, suggesting that Fur does not directly control *fpa* transcription.

What alters the ratio of Fur to Fpa? One hypothesis we will test is that apo-Fur association with Fpa results in a complex stabilizing Fpa. If Fur is not present, Fpa is degraded. We demonstrate that a Δ*fur* mutant accumulates cellular Fe and increases the pool of non-chelated Fe (Fig. 2). An alternate hypothesis is that the formation of a complex between Fe ions and Fpa causes a conformational change that targets Fpa for degradation, and apo-Fpa accumulates. In support of this hypothesis, we determined that *S. aureus* Fpa binds Fe(II). The internal concentration for Fe(II) in *S. aureus* cells is unknown, but it is predicted to be *≈*6 µM in aerobically cultured *E. coli* (47). Fpa binds Fe(II) with an affinity (*K*_D_ *≈* 2 µM) consistent with Fpa being holo and apo in Fe replete and deplete conditions, respectively. Moreover, it is currently unknown if the *S. aureus* Fur binds Fe(II) and, if so, what the affinity for Fe(II) is. It is tempting to speculate that *S. aureus* uses Fpa to help sense cytosolic non-chelated Fe concentration with apo-Fpa, but not holo-Fpa, imparting a regulatory effect upon Fur. Much more work is necessary to validate these hypotheses. Once we have gained a better understanding of the residues that promote interaction between Fur and Fpa, we can examine if Fur or Fpa variants with directed changes in these residues alter the abundance Fpa *in vivo* and examine the effect of Fe starvation on Fpa abundance. We also need to determine what effect the inhibition or removal of specific proteolysis machineries have on Fpa abundance in the WT strain upon Fe starvation.

Why would an anti-repressor be used by Firmicutes to relieve Fur-mediated repression? Bioinformatic analyses found that *fpa* was recruited to a *fur*-containing genome and was then retained long-term, apparently shaping the evolutionary trajectory of *fur*. It is likely that Fur found in *fpa*-containing organisms functions differently than in organisms that do not contain *fpa*. These same bioinformatic analyses failed to identify a specific Fur residue or a region that differs in genomes that do or do not code Fpa. A further in-depth study on the interactions between Fur and Fpa, including identifying and studying the residues driving these interactions, may shed light on why an anti-repressor role for Fpa evolved.

## MATERIALS AND METHODS

### Chemicals and growth conditions

All bacterial strains used in this study (Table 1) were derived from the community-associated methicillin-resistant *S. aureus* isolate USA300_LAC that had been cured of the pUSA3 plasmid coding erythromycin resistance (48). Bacteria were grown at 37°C in TSB (MP Biomedicals) and shaking at 200 rotations per minute. Solid media was generated by adding 1.5% (wt/vol) agar (VWR). Chelex-treated TSB was prepared as previously described (49). Unless stated otherwise, cells were cultured in 10 mL capacity culture tubes containing 1.5 mL of liquid medium. Quantitative growth was conducted using a 96-well plate reader BioTek 808E visible absorption spectrophotometer at 37°C and shaking. For quantitative growth, bacterial strains were grown for 18 hours and washed twice with phosphate-buffered saline (PBS) before diluting them to an optical density at 600 nm (OD_600_) of 0.02 in 200 µL. Anaerobic growth was conducted using a 37°C incubator inside of a Coy anaerobic chamber containing an oxygen scavenging catalyst to maintain oxygen levels <1 parts per million (ppm). Anaerobic growth was quantified by measuring culture OD_600_ after 18 hours.

**TABLE 1:** Bacterial strains and plasmids used in this study

Strain name	Genotype or function	Reference for allele or plasmid
*S. aureus* USA300_LAC strains		
JMB1100	USA300_LAC wild type (WT)	Alexander Horswill
JMB8689	Δ*fpa::tetM*	This study
JMB8428	*fpa::Tn*	(50) this study
JMB1432	Δ*fur::tetM*	(51)
JMB4536	*hemB::Tn*	(52)
JMB10638	Δfpa::tetM fur*	This study
JMB10675	*proC::Tn*	(50) and this study
JMB10677	Δ*fpa::tetM proC::Tn*	(50) and this study
JMB10676	fur* proC::Tn	(50) and this study
JMB10678	Δfpa::tetM fur* proC::Tn	(50) and this study
JMB7525	*fhuC::Tn*	(6)
JMB10664	Δ*fur::tetM fhuC::Tn*	
JMB10721	Δ*fpa::tetM fhuC::Tn*	(50) and this study
JMB10722	Δfpa::tetM fur* fhuC::Tn	(50) this study
JMB10641	*fpa::Tn*Δ*fur::tetM*	This study
JMB 9738	*geh*::*suf _hld* RBS*_lacZ*	(53)
*B. subtilis* strains		
BD630	*hisA1 leuA8 metB5*	(54)
BD8592	*hisA1 leuA8 metB5 P_hyperspank__yfp_fpa*	This study
BD8012	*hisA1 leuA8 metB5 P_hyperspank__gfp*	(55)
VCB102	*BD8592* D*fpa::pMutin4*	This study
VCB103	*hisA1 leuA8 metB5* D*fpa::pMutin4*	This study
Plasmids		
pEPSA5	Inducible expression complementation	(56)
pEPSA5_*fpa*	Inducible expression complementation	This study
pCM28	Native promoter complementation	(57)
pCM28_*fpa*	Native promoter complementation	This study
pJB38	Mutant generation	(58)
pJB38_Δ*fpa::tetM*	Mutant generation	This study
pDR111_YFP	Protein interaction studies	(59)
pED2143	Protein interaction studies	This study
pMutin4-*fpa*	Knockout construct	This study
pOS_*saeP1_gfp*	Transcriptional reporter	Shaun R. Brinsmade
pOS_*isdC_gfp*	Transcriptional reporter	This study
pOS_*fhuA_gfp*	Transcriptional reporter	This study
pOS_*sbnA_gfp*	Transcriptional reporter	This study
pOS_*saeP1_lacZ*	Transcriptional reporter	This study
pOS_*isrR_lacZ*	Transcriptional reporter	This study
pOS-1-p*lgt*	Genetic complementation *lgt* promoter	(60)
pOS-1-p*lgt*_*fur* pGEX-6P1_*fpa* pGEX-6P1_*fur*	Genetic complementation *lgt* promoter Expression of *fpa* Expression of *fur*	(2) This study This study
pSmBIT	Split luciferase assay	(61)
pLgBIT pSmBIT_*fpa*	Split luciferase assay Split luciferase assay	(61) This study
pSmBIT_*fur* pLgBIT_*fur*	Split luciferase assay Split luciferase assay	This study This study
pLgBIT_*fpa*	Split luciferase assay	This study

Streptonigrin sensitivity assays were performed as previously described (10). Overnight TSB cultures were washed with PBS and diluted to OD_600_ 0.1. One hundred microliter of cell suspension was added to 4 mL of TSA containing 0.35% agar. For genetic complementation studies, the TSA top agar also contained 1% xylose and 30  µg mL^−1^ chloramphenicol (Cm). Five microliter of 2.5 mg mL^−1^ of streptonigrin diluted in dimethyl sulfoxide (DMSO) was spotted atop the top agar overlay. The zone of inhibition was measured after overnight incubation at 37°C.

When necessary, antibiotics were added at the final following concentrations: 100 µg mL^−1^ ampicillin (Amp); 10 µg mL^−1^ Cm; 10 µg mL^−1^ erythromycin (Erm); 3 µg mL^−1^ tetracycline (Tet). Protein concentrations were determined using Bradford reagent (Bio-Rad Laboratories Inc., Hercules, CA). DNA primers were purchased from Integrated DNA Technologies and are listed in Table S8. Molecular reagents were purchased from New England Biolabs, unless otherwise stated. Unless stated otherwise, all chemicals were purchased from Sigma-Aldrich (St. Louis, MO). Sanger DNA sequencing was performed at Azenta (South Plainfield, NJ).

### Plasmid and strain construction

All plasmids used in this study are listed in Table 1. *E. coli* DH5α was used for plasmid preparation. The restriction minus strain *S. aureus* RN4220 was used for transformation (62), and transductions were carried out using bacteriophage 80α (63).

We used the pJB38 plasmid to create the Δ*fpa::tetM* mutant as previously described (64). Briefly, the chromosomal regions upstream and downstream of *fpa* were amplified by PCR using the following primer pairs: YCCylaNupFor and ylaNuptetRrev; tetRylaNdwnfor and ylaNdwnpJB38. We amplified *tetM* from strain JMB1432 using primers ylaNuptetRfor and tetRylaNdwnrev. The amplicons were gel purified and combined with pJB38 that had been digested with SalI and NheI. The fragments were combined using yeast recombinational cloning (65, 66). The pEPSA5_*fpa* vector was constructed by amplifying *fpa* from genomic DNA using the following primer pair: YlnA forEcoRI and YlnA revSalI. The pCM28_*fpa* vector was made by amplifying *fpa* from genomic DNA using the following primer pair: YlnA for5BamHI and YlnA revSalI. Amplicons and vectors were digested by EcoRI and SalI before ligation. To create the pGEX-6P-1_*fpa* expression vector, we amplified *fpa* from *S. aureus* using the following primer pair: ylaN5GSTBamHI and ylaN3XhoI. The amplicon was digested with BamHI and XhoI and ligated into similarly digested pGEX-6P-1. To create pGEX-6P1_*fur* expression vector, we amplified *fur* from *S. aureus* using the following primer pair: BamHI_fur_ATG_FWD and EcoRI_fur_REV. This was followed by restriction digest with BamHI and EcoRI and ligation into pGEX-6P1 with transformation into CopyCutter EP1400 *E. coli*. All plasmids were sequence verified.

All amplicons to create *gfp* expressing transcriptional reporters were cloned into similarly digested pOS_*saeP1_gfp*. To create the pOS_*isdC_gfp* transcriptional reporter plasmid, we amplified the *isdC* promoter using the following primer pair: pOS_iscC_kpnI and pOS_iscC_hindIII 5. For the pOS_*fhuA_gfp* transcriptional reporter plasmid, we amplified the *fhuA* promoter using the following primer pair: pOS_fhuA_hindIII 5 and pOS_fhuA_kpnI 3. For the pOS_*sbnA_gfp* transcriptional reporter plasmid, we amplified the *sbnA* promoter using the following primer pair: Sbn pro3 kpnI and Sbn pro5 hindIII.

To create the pOS_*saeP1_lacZ* transcriptional reporter, *lacZ* was amplified from strain JMB9738 using the following primer pair: lacZ TIR up kpnI and lacZ TIR down EcoRI. The digested PCR product was ligated into similarly digested pOS_*saeP1_gfp* plasmid creating pOS_*saeP1_lacZ*. To create pOS_*isrR_lacZ*, we amplified *isrR* from JMB1100 DNA using the following primer pair: HindIII tsr25p up and kpnI tsr25p dwn. The digested *isrR* amplicon was ligated into similarly digested pOS_*saeP1_lacZ* creating pOS_*isrR_lacZ*

To construct the *B. subtilis yfp_fpa* construct, *fpa* was amplified from *B. subtilis* BD630 genomic DNA (*hisA1 leuA8 metB5*), using the primers 5-yfp-ylaN and 3-yfp-ylaN. The vector pDR111-YFP was linearized by digestion with SalI and SphI. The In-Fusion HD cloning kit (Clontech) was used for cloning according to the manufacturer’s instructions. The resulting plasmid was transformed into Stellar competent cells (Clontech), isolated, and verified by DNA sequence analysis performed by Eton Bioscience (Union, NJ). The resulting plasmid (pED2143) was transformed into BD630 selecting for spectinomycin resistance, placing the *yfp-fpa* fusion under the control of the IPTG-inducible *P_hyperspank_* promoter at the *amyE* locus, creating the strain BD8592. The strain was confirmed by sequencing performed by Eton Biosciences.

To construct the strains necessary for the split luciferase assay, *fpa* was amplified from *S. aureus* using the following primer pair: YlaN_pSmBIT_pLgBIT_F and YlaN_pSmBIT_pLgBIT_R, and *fur* was amplified from *S. aureus* via the following primer pair: Fur_pSmBIT_pLgBIT_F and Fur_pSmBIT_pLgBIT_R. The vectors, pSmBIT and pLgBIT, were digested with KpnI and used as a template in PCR with the following primer pair: IM515 and IM1360. PIPE cloning (67) was used via mixing the *fpa* and *fur* amplicons with 50 ng linearized vector amplicons at a molar ratio of 7:1 (insert:vector), followed by transformation. All plasmids were sequence verified.

### Isolating and mapping suppressor mutations

Ten independent overnight cultures of the Δ*fpa::tetM* strain were cultured overnight in TSB. Cultures were individually diluted 1:100 into PBS, and 100 µL was plated onto TSA plates with 700 µM DIP. One colony from each of the 10 plates was taken and further characterized. The Δ*fpa::tetM* mutants with suppressor mutations had growth defects indicative of the strains having a null mutation in *fur*. We amplified the *fur* allele from the Δ*fpa::tetM* strain and each suppressor strain using the 1,448 veri 5 and 1,448 veri 3 primer pair. After gel purification, we sequenced the amplicons.

### *yfp_fpa* genetic complementation

To perform a complementation experiment, the native chromosomal copy of *fpa* must be inactivated, which is challenging because *fpa* is essential. In addition, *fpa* lies immediately upstream of another essential gene, *ftsW*. To avoid the disruption of downstream genes, *fpa* was cloned into the vector p*Mutin4* (68). A 250 bp internal fragment of *fpa* was amplified from BD630 genomic DNA using the primers 5 ylaN-pMutin4 and 3 ylaN-pMutin4. Following digestion, the fragment was ligated into the *Eco*RI and *Bam*HI sites of p*Mutin4*. The resulting plasmid (p*Mutin4_fpa*) was transformed into DH5α cells and verified by sequencing, performed by Eton Biosciences. This plasmid was used to transform BD630 and BD8592. p*Mutin4* will integrate into the *Bacillus* chromosome by Campbell integration with selection for erythromycin resistance, creating a knockout of the chromosomal *fpa* gene with the downstream genes under the control of the IPTG-inducible promoter *P_spac_*. Following transformation, BD630 and BD8592 cells were plated on lysogeny broth (LB) plates containing erythromycin or erythromycin and spectinomycin, respectively, with 0.5 mM FeCl_3_ and 0.5 mM IPTG, to induce downstream genes and *yfp_fbp* where appropriate. The following morning, individual colonies were struck on plates with and without FeCl_3_.

### RNA extraction, cDNA synthesis, and quantitative PCR (qPCR)

*S. aureus* strains were cultured overnight in TSB and subsequently diluted in triplicate to an OD_600_ of 0.1 in 2.5 mL TSB with or without 500 µM DIP in 10 mL glass culture tubes. Cells were incubated at 37°C with agitation for 6 hours after which time 1 mL cell pellets were treated with RNAprotect (Qiagen). Cell pellets were washed in 0.5 mL PBS pH 7.4, resuspended in 100 µL 50 mM tris pH 8 containing 6.7 µg lysostaphin, and then incubated at 37°C with agitation for 30 minutes. The cell suspension was incubated at 65°C for 5 minutes following the addition of 200 µL of 20 mM sodium acetate, 1 mM EDTA, 0.5% SDS with 13.4 µg lysostaphin. RNA isolation was performed as previously described (24). cDNA libraries were constructed using the High-Capacity cDNA Reverse Transcription kit (Biosystems). Quantitative real-time PCR was performed using an Applied Biosystems StepOnePlus thermal cycler. Data were analyzed using the comparative C_T_ method (49, 69).

### CAS siderophore assay

Overnight cultures in TSB were diluted 100-fold into 1 mL of Chelex (Bio-Rad)-treated TSB with the addition of 25 µM zinc acetate, 25 µM MnCl_2_, 1 mM MgCl_2_, and 100 µM CaCl_2_ in 10 mL glass culture tubes. The cultures were incubated at 37°C with shaking for 18 hours. The chrome azurol S siderophore assay was performed on the spent media using the modified microplate method as previously reported (70, 71).

### Whole cell metal quantification

*S. aureus* strains were grown for 18 hours overnight in TSB before diluting them to an OD of 0.05 (A_600_) in 7.5 mL of Chelex (Bio-Rad)-treated TSB in a 30 mL capacity culture tubes as described previously (49). Cells were allowed to grow with shaking for 8 hours. Pre-weighted metal-free 15 mL propylene tubes were used to pellet the cells using a prechilled tabletop centrifuge (Eppendorf, Hauppauge, NY). Pellets were washed three times with 10 mL of ice-cold PBS. All samples were kept at −80°C or on dry ice until processing.

Cell pellets were acid digested with 2 mL of Optima grade nitric acid (ThermoFisher, Waltham, MA) and 500 µL hydrogen peroxide (Sigma, St. Louis, MO) for 24 hours at 60°C. After digestion, 10 mL of UltraPure water (Invitrogen, Carlsbad, CA) was added to each sample. Elemental quantification on acid-digested liquid samples was performed using an Agilent 7700 inductively coupled plasma mass spectrometer (Agilent, Santa Clara, CA). The following settings were fixed for the analysis Cell Entrance = −40 V, Cell Exit = −60 V, Plate Bias = −60 V, OctP Bias = −18 V, and collision cell Helium Flow = 4.5 mL min^−1^. Optimal voltages for Extract 2, Omega Bias, Omega Lens, OctP RF, and Deflect were determined empirically before each sample set was analyzed. Element calibration curves were generated using ARISTAR ICP Standard Mix (VWR). Samples were introduced by a peristaltic pump with 0.5 mm internal diameter tubing through a MicroMist borosilicate glass nebulizer (Agilent). Samples were initially uptaken at 0.5 rps for 30 s followed by 30 s at 0.1 rps to stabilize the signal. Samples were analyzed in Spectrum mode at 0.1 rps, collecting three points across each peak and performing three replicates of 100 sweeps for each element analyzed. The sampling probe and tubing was rinsed for 20 s at 0.5 rps with 2% nitric acid between each sample. Data were acquired and analyzed using the Agilent Mass Hunter Workstation Software version A.01.02.

### Transcriptional reporter assays

*S. aureus* strains were grown overnight in 2 mL TSB supplemented with 10 µg mL^−1^ chloramphenicol in 10 mL culture tubes at 37°C with shaking. *S. aureus* cultures were then subcultured 1:100 into 5 mL TSB supplemented with 10 µg mL^−1^ chloramphenicol ±500 µM DIP in 10 mL culture tubes and incubated at 37°C with shaking for 8 hours. For strains containing *gfp* expression reporters, OD_600_ and GFP fluorescence (excitation 485 nm and emission 520 nm) were measured using a Varioskan Lux plate reader (Thermo Scientific). GFP fluorescence relative to OD_600_ was determined. The assays were performed using biological quintuplets. For strains containing the pOS_*isrR*_*lacZ* expression reporter, beta-galactosidase activity was measured, and modified Miller Units were calculated as reported previously (53). The assay was performed using biological triplicates.

### Bioinformatic analyses

The predicted protein sequences from all assembled bacterial genomes designated as “reference” or “representative” in the national center for biotechnology information (NCBI) database were retrieved on 14 March 2023. The *S. aureus* Fpa sequence (ABD22267.1) was used as a query for a DIAMOND (v2.1.2; “--ultra-sensitive --max-target-seqs 0”) (72) search against the bacterial proteins retrieved from NCBI. Only hits with an *e*-value <1e^−5^ were retained for downstream analysis; bacterial genomes that encode proteins with hits (*e*-value <1e^−5^) to the Fpa query were considered Fpa-positive. Four Fur sequences (ABD21033.1, P54574, P0C6C8, and P0A9A9), two from the species of interest in this study and two from species known to not encode Fpa, were used as queries for a DIAMOND (v2.1.2; “--ultra-sensitive --max-target-seqs 0”) search against the bacterial proteins retrieved from NCBI. Only hits with an *e*-value <1e^−5^ were retained for downstream analysis. The hits from each of the four Fur query sequences were parsed separately, retaining the best (highest scoring) hit per genera. The top-scoring sequences (one per genera in the output) from each of the queries were combined and had redundant sequences (those identified by multiple queries) removed. The combined non-redundant sequences were aligned using mafft (v7.453; “--localpair --maxiterate 1,000”) (73), with the resulting alignment used by fastme (v2.1.5) (74) for phylogenetic inference. Gotree (v0.4.4) and Goalign (v0.3.6) (75) were used for file format conversion between analysis steps, and iTOL (76) was used for visualization. The filtering approach (down sampling to genera) applied to the Fur hits was performed to reduce the number of sequences to an amount that is computational tractable for phylogenetic analysis (i.e., it reduced the number of sequences in the resulting phylogeny from 10’s of thousands to only a few thousand).

The sequences that form the large clade of Firmicutes Fur sequences (Fig. 5) were extracted for reanalysis. The protein sequences were realigned using mafft (“--localpair --maxiterate 1,000”) and had a phylogeny inferred using iqtree (v1.6.12; “-m MFP -bb 2,000”). The alignment and phylogeny were compared together visually using iTOL. To improve readability, a phylogeny with a reduced number of taxa (10% chosen at random from the full phylogeny) was also constructed and visualized using the same approach (Fig. S6). A phylogeny of the Fur sequences from genomes which encode Fpa sequences (Fpa-positive genomes) and a phylogeny of the Fpa sequences from these genomes were inferred from the Firmicutes extracted from Fig. 5, with mafft and iqtree used for alignment and phylogeny inference (using the same versions and parameters as previously stated). Fur and Fpa sequences from genomes with multiple *fur* or *fpa* genes identified (using the previously described DIAMOND search results) were removed to prevent problems arising from paralogous sequences. The Fur and Fpa trees were compared using the tanglegram function (“sort = TRUE and rank_branches = TRUE”) from the dendextend (v1.17.1) R package (77).

To map changes in gene expression to the KEGG pathway maps, the predicted proteins for *S. aureus* subsp. USA300_FPR3757 were downloaded from NCBI (NC_007793) and assigned Kyoto Encyclopedia of Genes and Genomes (KEGG) Orthology (KO) numbers using the KEGG Automatic Annotation Server [gene data set: *S. aureus* USA300_FPR3757 (saa), search tool: BLAST] (78). The NCBI gene IDs were manually assigned to the old *S. aureus* gene names used for expression analysis and used to transfer KO number annotations.

### Fpa interaction experiments

#### Immunopurification of YFP-Fpa

*B. subtilis* strain BD8592 and BD8012 (GFP only control) were grown overnight in 50 mL of LB with 100  µg mL^−1^ spectinomycin. After incubation, cells were diluted 1:100 (vol/vol) into 2 L of fresh LB media with the addition of 0.5 mM IPTG and grown for 1.5 hours for exponential phase or 4 hours for stationary phase. Growth was monitored hourly by the optical density at OD_600_. Cells were harvested by centrifugation, frozen as pellets in liquid nitrogen, and subjected to cryogenic cell lysis as described previously (33). A total of 0.75 g of frozen cell powder was immediately added to 10 mL of lysis buffer [20 mM HEPES, pH 7.4, 100 mM potassium acetate, 2 mM MgCl_2_, 0.1% tween-20 (vol/vol), 1 µM ZnCl_2_, 1 µM CaCl_2_, 0.25% Triton-X, 200 mM NaCl, 1:100 protease inhibitor cocktail (Sigma), and 0.1 mg mL^−1^ phenylmethylsulphonyl fluoride]. The resulting suspension was homogenized for 20 s using a PT 10–35 GT Polytron (Kinematica) and centrifuged for 10 minutes at 8,000 × *g* at 4°C. The soluble fraction was mixed with 10 µL of GFP-Trap magnetic agarose (Proteintech) for 1  h with gentile rotation at 4°C. The magnetic beads were recovered and washed four times with lysis buffer without inhibitors and two times with PBS. Proteins were eluted directly into 50 µL of TEL buffer (106 mM Tris-HCl, 141  mM Tris-base, 0.5 mM EDTA, 2.0% lithium dodecyl sulfate , and pH 8.5) for in-solution digestion (79, 80).

#### Preparation of samples for mass spectrometry

Samples were alkylated and reduced with final concentrations of 30 mM chloroacetamide and 10 mM tris(2-carboxyethyl)phosphine hydrochloride (TCEP, pH 7.0) and heated to 95°C for 5 minutes. Protein digestion was performed using a filter-aided sample preparation method (81–83). Briefly, filters [Vivacon 500 centrifugal filters (10K cut-off), Sartorius Stedim Biotech, Goettingen, Germany] were used for desalting and overnight trypsin digestion at 37°C with a 1:50 enzyme to protein ratio in 40 mM HEPES, pH 7.4, and 0.1% sodium deoxycholate. Following digestion, the detergent was removed from the samples through acidification with 1% triflouroacetic acid (final concentration) as described (55, 84). Soluble peptides were desalted using SDB-RPS Stage Tips and eluted in 50 mL of 5% ammonium hydroxide-80% acetonitrile (ACN) (85). Samples were evaporated to near dryness by vacuum centrifugation and resuspended in 1% formic acid (FA)/4% ACN to bring the total volume to 10 mL.

#### Mass spectrometry

Samples were analyzed by nano-liquid chromatography-tandem mass spectrometry (nLC-MS/MS) on a Dionex Ultimate 3000 RSLC coupled directly to an LTQ-Orbitrap Velos mass spectrometer (ThermoFisher Scientific, San Jose, CA), through a Nanospray Flex ion source (ThermoFisher Scientific). Approximately 4 µL of peptides were directly injected for analysis. Instrument parameters and settings were as described previously (86), and except peptides were separated using a 150 minutes linear reverse phase gradient. The mass spectrometer was operated in a data-dependent acquisition mode with each cycle of analysis containing a single full-scan mass spectrum (*m*/*z* = 350–1,700) in the orbitrap (*r* = 120,000 at *m*/*z* = 400) followed by collision-induced dissociation MS/MS of the top 15 most abundant ions, with dynamic exclusion enabled.

#### Informatics workflow

The MS/MS spectra were extracted using the Proteome Discoverer software platform (ver. 1.4, Thermofisher Scientific), and spectra were analyzed using SEQUEST (ver. 1.4.0.288. ThermoFisher Scientific) and for database searching against the UniProt SwissProt sequence database (downloaded 04/2017) composed of *B. subtilis* and *E. coli* reference proteome sequences, including common contaminant sequences (total of 8,604 sequences). The processing workflow parameters were defined as follows: peptides with at least six amino acids, full trypsin cleavage specificity, and up to two missed cleavages. Database searching was performed with precursor and fragment ion mass tolerances of 10 ppm and 0.4 Da, respectively. Cysteine carbamidomethylation was denoted as a fixed modification and methionine oxidation, asparagine deamidation, phosphorylation of serine and threonine, lysine acetylation, and n-terminal acetylation, as variable modifications. Peptide and protein identifications were validated using Scaffold (ver. 4.8.1, Proteome Software, Inc.), with a peptide and protein false discovery rate (FDR) threshold of <1.0% and a minimum of two unique peptides in at least one condition. X! Tandem [www.thegpm.org, ver. CYCLONE (2010.12.01.1)] was set up to search a reverse concatenated subset of the same database. For LFQ, spectral counts were used to determine enrichment vs the negative GFP controls, while precursor intensity, based on the sum of the three highest intensity peptides for each protein (T3PQ), was used for comparing interaction abundance in log vs stationary phases (87).

### Split luciferase assay

Interaction between *S. aureus* Fur and Fpa was assessed *in vivo* in an *S. aureus* strain lacking Fur and Fpa (Δfpa::tetM fur* proC::Tn JMB10678) using a split luciferase system. Overnight cultures (2 mL in 10 mL glass culture tubes) grown at 37°C with agitation in TSB supplemented with chloramphenicol (10 µg mL^−1^) and kanamycin (50 µg mL^−1^) were diluted 1:10 into 1 mL fresh TSB in a 10 mL glass culture tube supplemented with chloramphenicol and kanamycin and were incubated for 6 hours at 37°C with agitation. These cultures were then diluted 1:100 into fresh TSB supplemented with chloramphenicol, kanamycin, anhydrotetracycline (5 ng µL^−1^), and Nano-Glo Luciferase Assay Substrate (1:1,000; Promega) (61). The cultures were dispensed in triplicate into a sterile 96-well black well clear bottom plate. The plate was incubated at 37°C with shaking at 300 rpm in between measurements in a Varioskan Lux microplate reader. Culture OD_600_ and light emission (1 second exposure) was measured after 30 minutes.

### Fpa and Fur purification

*E. coli* strain BL21(DE3) containing pGEX-6P-1_*fpa* was cultured in 3 L of 2× LB supplemented with ampicillin (50 µg mL^−1^) at 30°C with shaking. After reaching an OD_600_ of 0.6, the temperature was shifted to ~25°C, and *fpa* expression was induced with 100 µM IPTG for ~16 hours. The cells were harvested by centrifugation and resuspended in purification buffer (150 mM NaCl, 50 mM Tris-HCl, and pH 7.5) containing 1% wt per volume lysozyme. Cells were disrupted using sonication, and cell debris was removed by centrifugation. The cell free extract was filtered through a 0.45 µM filter before loading into a 5 mL GSTrap FF column (GE Healthcare).

The GSTrap FF column was equilibrated with purification buffer, and filtered extract was loaded onto the column. The column was washed with 40 column volumes of purification buffer. After washing, the column was incubated with one column volume of cleavage buffer (50 mM Tris-HCl, pH 7.0, 150 mM NaCl, 1 mM EDTA, and 1 mM dithiothreitol) containing 500 units PreScission Protease (Cytiva). Following overnight incubation, Fpa was eluted using 5 mL of cleavage buffer. Eluted protein was concentrated using spin concentrators (Millipore Amicon Ultra-15–3 K) to ~1.5–2 mL and subsequently dialyzed in 1 L of storage buffer [150 mM NaCl, 50 mM Tris-HCl, pH 7.5, and 5% (vol/vol) glycerol] three individual times to remove EDTA. Fpa was flash frozen and stored at −80°C until use. Protein concentration was measured using a Bradford assay.

For Fur purification, *E. coli* strain BL21(DE3) containing pGEX-6P1_*fur* was cultured in the same manner as described above for the purification of Fpa. Fur was purified in the same manner as described above for the purification of Fpa with the exception that the storage buffer that Fur was dialyzed into after purification also contained 1 mM TCEP.

### Electrophoretic mobility shift assay

Binding between Fur and the *isdC* operator region was assessed using an EMSA. The 128 base pair *isdC* operator region was amplified using the following primer pair: isdC_2FurBox_EMSA_F and isdC_2FurBoxEMSA_R. Purified Fur protein (0–80 µM) incubated for 20 minutes at room temperature in binding buffer [20 mM tris pH 8, 1 mM MgCl_2_, 50 mM KCl, 5% (vol/vol) glycerol, 100 µM MnCl_2_, and 1 mM dithiothreitol prior to the addition of 0.0625 µM *isdC* operator DNA (88). The reaction incubated at room temperature for another 30 minutes prior to the addition of 10× loading dye [binding buffer, 30% (vol/vol) glycerol, and 0.1% bromophenol blue]. The samples were loaded onto a pre-run (120 V at 4°C for 2 hours) 8% polyacrylamide tris borate native PAGE gel and run at 120 V at 4°C for 1.75 hours. The gel was stained using Thermo Fisher Scientific EMSA kit with Sybr Green following the manufacturer’s protocol.

A titration of purified Fpa protein (0–80 µM) into binding reactions containing 40 μΜ Fur was performed to assess the concentration of Fpa at which Fur binding to the *isdC* operator is inhibited. The binding reaction was performed in the same manner as in the Fur titration, except for the addition of Fpa (0–80 µM) containing 100 µM MgCl_2_ into the binding reaction at the time of DNA addition. The EMSA was performed in the same manner as in the Fur titration EMSA.

### Proteomics

#### Samples preparation

Biological triplicate samples were grown in 2 mL of TSB, incubated at 37°C overnight with shaking. After this time, cells were subcultured to an OD_600_ of 0.1 in 2.5 mL fresh TSB ±500 µM DIP in 10 mL glass culture tubes and incubated for 6 hours at 37°C with shaking. Cell pellets were first washed with PBS and then resuspended in PBS containing 100 µg/mL lysostaphin, 100 U/mL DNAsel (Thermo Fisher), 25 U/mL RNAsel (Thermo Fisher), and 2× EDTA-free protease inhibitor cocktail (Pierce, Thermo Fisher) and transferred to a new 2 mL vessel with silica lysis beads. Cell suspensions were then incubated for 30 minutes in a 37°C water bath before cells were lysed via bead beating. Lysates were then added to cell lysis buffer with a final concentration of 5% (wt/vol) SDS, 50 mM tetraethylammonium bromide , pH 8.5, and 20 mM DTT. Samples were incubated at 95°C for 10 minutes and then centrifuged at 17,000 × *g* for another 10 minutes. The resulting supernatant, now free of any non-solubilized proteins, was quantified using the Pierce 660 nm assay with ionic detergent compatibility reagents (Thermo Fisher Scientific). Samples were standardized to 50 µg and then incubated with 40 mM iodoacetamide in the dark for 30 minutes at room temperature. The reaction was quenched with phosphoric acid to a final concentration of 1.2% in solution and then added in a 1:7 volumetric ratio to S-trap buffer [10% (vol/vol) TEAB pH 7.5, 90% (vol/vol) MeOH]. Samples were bound to the S-trap mini column (Protifi) via a series of repeated centrifugations at 4,000 × *g* for 30 seconds. A series of three washes were then performed with the S-trap buffer at the same centrifugation settings. S-trap columns were transferred to a new vessel followed by the addition of 50 mM TEAB digestion buffer containing trypsin/lys-C. The digestion buffer was added in a 1:10 ratio of protein:enzyme and incubated overnight at 37°C. Peptides were then eluted using 50 mM TEAB, 0.2% formic acid, and 50% acetonitrile in a series of three centrifugation steps at 4,000 × *g* for 30 seconds using each respective buffer. Following transfer to a vacuum centrifuge, samples underwent evaporation until complete dryness. Samples were then desalted using the SepPak C18 columns (Waters) according to manufacture instructions and were again vacuum centrifuged until dry. Samples were stored at 4°C prior to mass spectrometric analysis.

#### Mass spectrometry

Samples were resuspended in 50 µL of 0.1% formic acid with 5 µL being used for injection. Peptides were separated using a Ultimate3000 UHPLC (Thermo Fisher Scientific) on a 50 cm Acclaim PepMap 100 C18 reversed-phase high-pressure liquid chromatography column (Thermo Fisher Scientific) with a 180 minutes gradient (2%–32% acetonitrile with 0.1% formic acid). Subsequent peptide detection and analysis were performed on a hybrid Quadrupole-Orbitrap instrument (Q Exactive Plus; Thermo Fisher Scientific). The topmost abundant ions were selected for MS/MS analysis using data-dependent acquisition.

#### Data analysis

Raw files were searched using the MaxQuant proteomics platform (v1.6.3.4) with the integrated andromeda search engine. Files were referenced against the *S. aureus* USA300 pan proteome (UniProt ID: UP000001939) for peptide identification. Digestion was set to specific using the trypsin/lys-C enzyme. Fixed modifications included methionine oxidation and protein N-terminal acetylation, while variable modifications included cystine carbamidometylation. Peptides were identified with an FDR of less than 1%. Additional processing of the protein groups output file was performed using the Perseus software (v1.6.15.0). Contaminants were filtered out, and proteins considered for analysis were those with peptides found in at least two of the three biological replicates in any one condition and/or strain of *S. aureus*. LFQ intensities were Log2 transformed to facilitate statistical testing using the Welch’s *t*-test for determination of significant protein abundance changes. The mass spectrometry proteomics data have been deposited to the ProteomeXchange Consortium via the PRIDE (89) partner repository with the data set identifier PXD051930.

### Metal-binding competition assay

The binding affinity of Fpa for Fe(II), Zn(II), Mn(II), and Mg(II) was measured as previously reported (39). Briefly, this assay exploits Mag-Fura-2 (Molecular Probes) as a metal chelator chromophore to measure the binding of ferrous ions to an additional biomolecule (i.e., Fpa) under complex heterogeneous conditions. Mag-Fura-2 forms a 1:1 complex with Fe(II), Zn(II), Mn(II), and Mg(II) and displays a maximum absorbance at 325 nm when metal is bound; this is in contrast to a feature at 365 nm when the chelator is in the apo state (39, 40, 42). The transition of the absorbance feature at 365 nm, observed during metal loading of Fpa, was measured using a Shimadzu UV-1800 spectrophotometer housed within a Coy anaerobic chamber. Titration data for adding a divalent metal ion into the protein/Mag-Fura-2 mixture were collected anaerobically at room temperature using a 1 cm quartz cuvette. All spectra were collected anaerobically in 20 mM Tris and 150 mM NaCl (pH 7.5) buffer.

Samples were prepared for analysis in the following manner. Reagents/buffers were Ar(g) purged and equilibrated overnight within a Coy anaerobic wet chamber prior to experimentation. Holo- and apo-protein solution samples were prepared immediately before use under the same buffer and maintained under anaerobic conditions. Independent protein samples, incubated in 5 mM TCEP prior to titrations, were dialyzed using anaerobic buffer before analysis to remove the TCEP. Mag-Fura-2 concentrations were varied between 1 and 4 µM, while the protein concentration was held constant at 2 µM. A solution of 2 mM ammonium ferrous sulfate hexahydrate, zinc sulfate heptahydrate, manganese chloride tetrahydrate, and magnesium chloride were prepared in the anaerobic buffer listed previously and added in progressive increments until absorption saturation was reached. After each addition of aqueous metal ions, an absorption spectrum was collected between the wavelength of 200–800 nm. Initial apo Mag-Fura-2 concentrations were determined using the molar absorptivity (ε) value of 29,900 M^−1^cm^−1^ for the compound, measured at the wavelength of 365 nm (38). The absorbance at 365 nm, corrected for dilution, was then used to calculate binding parameters. Binding data were simulated with the program DYNAFIT (90), using a non-linear least squares analysis script to identify the binding capacity and metal stoichiometry in a manner previously outlined (38, 40). Each titration experiment was simulated using both one and two-site metal-binding models.

### Circular dichroism spectroscopy

Apo- and holo-Fpa samples were characterized for secondary structure content using a Jasco 1500 spectrophotometer as described previously (40). Briefly, samples were prepared anaerobically in a Coy wet chamber, loaded into a 0.1 cm quartz cuvette, and then transferred to the nitrogen purged sample chamber of the instrument where data were collected at 27°C. Spectra were collected on 5–10 µM protein concentration samples 5 mM NaPO_4_ buffer solutions at pH 7.5. For reproducibility, an average of 30 scans were collected on three samples per state, and results were averaged for final analysis. Before spectral collection, a baseline at each wavelength was subtracted to eliminate buffer signal. Data were analyzed using the Jasco CD Pro analysis software and simulated using the CONTIN method including the SP29, SP37, SP43, SMP50, and SMP56 reference sets (41). Values obtained from simulations using each database were averaged to obtain final analysis parameters.

### Differential scanning calorimetry

Thermal stability parameters for apo- and holo-Fpa were evaluated using a VP Differential Scanning Calorimeter (TA Instruments) housed anaerobically in a Coy wet chamber. Protein and Fe solutions were prepared anaerobically in 20 mM Tris and 150 mM NaCl solution at a pH of 7.5. Buffer was independently degassed with argon prior to sample preparation and loading. Protein concentrations between 3 and 5 mg mL^−1^ were used, and a 2.0 mM ferrous ion solution was added to obtain samples up to a 1:1 protein to Fe ratio at a final volume of 600 µL. DSC scans were run at a rate of 1°C per minute over a temperature range of 10°C–90°C. Pressure was maintained above 2.95 atm throughout the scan. Data were analyzed using the Nano Analyze software provided by TA Instruments. Baseline subtraction of buffer alone or buffer with Fe at concentrations that matched the protein samples was measured as controls. Peak deconvolution of the protein spectra was achieved using the two-state scaled mathematical model that incorporates the A_w_ factor, which accounts for inaccuracies in protein concentration upon denaturation. Data presented represent an average of three sample results.

### X-ray absorption spectroscopy

Fe(II)-Fpa samples were prepared in 20 mM Tris and 150 mM NaCl. The sample was brought to a 30% glycerol concentration for cryo-protection, and samples were flash frozen and stored in liquid nitrogen immediately after loading into prewrapped 2 mm Leucite XAS cells until exposed to the beam. Fe XAS was collected at the Stanford Synchrotron Radiation Lightsource on beamline 7–3. This beamline is equipped with a Si[220] double crystal monochromator with a mirror present upstream for focusing and harmonic rejection. We used a Canberra 30 element germanium solid state detector to measure protein fluorescence excitation spectra. Temperature during collection was maintained at 10 K by an Oxford Instruments continuous-flow liquid helium cryostat, and data were collected with the solar slits utilizing a 3 µm Mn filter placed between the cryostat and detector to diminish background scattering. XAS spectra were collected in 5 eV increments in the pre-edge region, 0.25 eV increments in the edge region, and 0.05 Å^−1^ increments in the EXAFS region to k = 14 Å^−1^, integrated from 1 to 25 s in a k^3^-weighted manner for a total scan length of ∼40 minutes. Fe foil absorption spectra were simultaneously collected with each respective protein run and used for calibration and assigning first inflection points for the metals (7111.2 eV)

XAS spectra were processed and analyzed using the EXAFSPAK program suite written for Macintosh OS-X (91), integrated with the Feff v8 software (92) for theoretical model generation. Normalized XANES data were subjected to edge analysis for both metals and in the case of Fe to pre-edge analysis as well. The Fe 1s-3d pre-edge peak analysis was completed as described previously (93); peak area was determined over the energy range of 7,110–7,116 eV. Oxidation state was deduced from the first inflection energies of the respective edges (42). Data were collected to *k* = 14 Å^−1^, which corresponds to a spectral resolution of 0.121 Å^−1^ for all metal–ligand interactions (42); therefore, only independent scattering environments at distances >0.121 Å were considered resolvable in the EXAFS fitting analysis. Data were fit using both single and multiple scattering model amplitudes and phase functions to simulate Fe-O/N, -S, and -Fe ligand interactions. During Fe data simulations, a scale factor (Sc) of 0.95 and threshold shift (ΔE_0_) value of −10 eV (Fe–O/N/C), −12 eV (Fe–S), and −15 eV (Fe–Fe) were used. These values were obtained from fitting crystallographically characterized small molecule Fe (93). The best-fit EXAFS simulations were based on the lowest mean square deviation between data and fit, corrected for the number of degrees of freedom (*F’*) (94). During the standard criteria simulations, only the bond length and Debye-Waller factor were allowed to vary for each ligand environment.

## SUPPLEMENTAL MATERIAL

Supplemental material mbio.02310-24-s0001.pdf Supplemental tables and figures.

Table S1 mbio.02310-24-s0002.xlsx Protein interaction data.

Table S2 mbio.02310-24-s0003.xlsx Proteomics data.

ASM does not own the copyrights to Supplemental Material that may be linked to, or accessed through, an article. The authors have granted ASM a non-exclusive, world-wide license to publish the Supplemental Material files. Please contact the corresponding author directly for reuse.
