# Supplementary material for: Fpa (YlaN) is an iron(II) binding protein that functions to relieve Fur-mediated repression of gene expression in Staphylococcus aureus
Source: mBio. 2024 Oct 23;15(11):e02310-24. doi: 10.1128/mbio.02310-24 (PMC11559061; doi:10.1128/mbio.02310-24)
Supplement: Supplemental material — Supplemental tables and figures. [file mbio.02310-24-s0001.pdf]

Supplemental material for:

Fpa (YlaN) is an iron(II) binding protein that functions to relieve Fur-mediated repression of gene expression in *Staphylococcus aureus*.

Jeffrey M. Boyd[1*], Kylie Ryan Kaler[1], Karla Esquilín-Lebrón[1], Ashley Pall[2], Courtney J. Campbell[2], Mary E. Foley[1], Gustavo Rios[1], Emilee M. Mustor[6], Timothy G. Stephens[1], Hannah Bovermann[1], Todd M. Greco[3], Ileana M. Cristea[3], Valerie J. Carabetta[4], William N. Beavers[$5], Debashish Bhattacharya[1], Eric P. Skaar[5], Lindsey N. Shaw[6], Timothy L. Stemmler[2*]

[1]Department of Biochemistry and Microbiology, Rutgers, the State University of New Jersey, New Brunswick, NJ, USA.
[2]Department of Pharmaceutical Sciences, Wayne State University, Detroit, MI, USA.
[3]Department of Molecular Biology, Princeton University, Washington Road, Princeton, NJ, USA.
[4]Department of Biomedical Sciences, Cooper Medical School of Rowan University, Camden, NJ, USA.
[5]Department of Pathology, Microbiology, and Immunology, Vanderbilt University Medical Center, Nashville, TN, USA.
[6]Department of Molecular Biosciences, University of South Florida, Tampa, FL, USA.

[$]Present address: Department of Pathobiological Sciences, Louisiana State University, Baton Rouge, Louisiana, USA.

*To whom correspondence may be addressed:
Jeffrey M. Boyd, Department of Biochemistry and Microbiology, Rutgers University, 76 Lipman Drive, New Brunswick, New Jersey, 08901, Telephone: (848) 932-5604, E-mail: jeffboyd@SEBS.rutgers.edu.

Timothy L. Stemmler, Department of Pharmaceutical Sciences, Wayne State University, 259 Mack Avenue, Detroit, Michigan, 48201, Telephone: (313) 577-5712, E-mail: tstemmle@med.wayne.edu.

Table S3. Binding affinities of Fpa for Zn(II) and Mn(II). Summary of the binding affinities for Zn(II) and Mn(II) using a two site binding model to measure metal affinity at each independent binding site.

Metal	K_{d1} (M)	K_{d2} (M)
Zn(II)	1.36E-07 ± 0.01E-07	1.83E-08 ± 0.14E-08
Mn(II)	2.51E-07 ± 0.68E-07	1.50E-07 ± 0.33E-07

Table S4. Circular Dichroism Simulation Parameters for Apo- and Holo- Fpa. Secondary structure elements tested include α-helix, β-sheet, alternate structured (turns, loops, etc.), and unstructured.

Sample	Secondary Structure				RMSD [a]
	α- Helix	*β*- Strand	Alt. Str.	Unstructured	
**Fpa**	31.3 ± 4.6%	18.6 ± 3.3%	20.7 ± 0.4%	29.4 ± 1.0%	0.09
**Fpa+Fe(II)**	40.1 ± 3.2%	16.0 ± 5.2%	18.8 ± 1.9%	25.1 ± 1.5%	0.03

[a] Root Mean Squared Deviation between empirical and theoretical spectrum from the simulation.

Table S5. Differential Scanning Calorimetry Thermodynamic Stability Parameters for Apo- and Holo- Fpa.[a]

Metal State	Tm*fit1* (°C)	Tm*fit2* (°C)	ΔH*cal* (kJ/mol)	ΔS*cal* (J/mol•K)
**Apo**	72.02 (±0.93)	77.49 (±0.55)	25.9	74.0
**Holo**	75.26 (±0.25)	79.80 (±0.04)	30.5	86.1

[a]Tm*fit1* and Tm*fit2* represent individual melting temperatures from each transition in the raw data simulation. ΔH*cal* and ΔS*cal* represent enthalpy and entropy values obtained from peak integration after baseline correction of raw empirical data.

Table S6. Analysis of Pre-Edge and Edge First-Inflection Point Features from Fe-XANES from Fpa Pre-edge analysis of the 1s→3d transition in Fe(II) Fpa samples. Peak inflection values were calculated by taking the first derivate of the edge feature for each.

Sample	First Inflection Edge Energy (eV)	Pre-Edge Area [a]
**Fpa**	7123.0 (±0.05)	0.024 (±0.002)

[a] Peak areas given in increments of eV^2.

Table S7. Summary of Fpa Fe-EXAFS Best Fit Simulation Parameters.

Spectra were fit over a *k*-range of 1.0-12.5 k^{-1}.

Nearest Neighbor Ligands [a]				Long Range Ligands [a]				
**Atom [c]**	**R (Å)[d]**	**C.N [e]**	**σ^2 [f]**	**Atom [c]**	**R (Å)[d]**	**C.N [e]**	**σ^2 [f]**	**F' [g]**
O/N	2.15	6.0	4.56	C	3.49	1.5	3.23	0.33
				C	4.15	1.5	2.76	

[a]/[b]- Independent metal-ligand scattering environment.
[c] - Scattering atoms: N (nitrogen), O (oxygen), C (carbon), S (sulfur).
[d] - Average metal-ligand bond length.
[e] - Average metal-ligand coordination number.
[f] - Average Debye-Waller factor (Å x 10^3).
[g] - Number of degrees of freedom weighted mean square deviation between data and fit.

Table S8. DNA primers used

Primer name	Primer sequence
ylaNdwnpJB38	TGAAATCAGAGCTTGCATGCCTGCAGGTCGACCCTGTAAGTTCATAAATCCTAAGTTT
tetRylaNdwnfor	GTATATAAACATTCTCAAAGGGATTTCTAAACGCGTGGCGTGAGATATCGACAATTG
ylaNuptetRfor	ATTTATATAGAGGGGGAGCGTGTCACGCGTCGGATTTTATGACCGATGATGAAGAAA
tetRylaNdwnrev	CTCAATTGTCGATATCTCACGCCACGCGTTTAGAAATCCCTTTGAGAATGTTTATATA
YCCCylaNupfor	ATAGCGTAACTATAACGGTCCTAATGTGCGCTAGCCCTCAATATGAGTTACATGGTT
ylaAuptetRrev	CTTTTCTTCATCATCGGTCATAAAATCCGACGCGTGACACGCTCCCCCTCTATATAA
G+tet nheI	CCCGCTAGCCGGATTTTATGACCGATGATGAAG
G+tet mluI	CCCACGCGTTTAGAAATCCCTTTGAGAATGTTT
YlnA forEcoRI	GGGGAATTCGAGGGGGAGCGTGTCATGGCGAAACAAGCAACA
YlnA revSalI	GGGGTCGACCGGCGCAGCTCAATTGTCGATATCTCACGCC
YlnA for 5BamHI	GGGGGATCCCCCACTCCCTATCAATGTATATAGC
5 ylaN-pMutin4	TGGTGGGAATTCGTTGACCATCGGCAAAAAGC
3 ylaN-pMutin4	CCACCAGGATCCCGCGTCATGCAAAGCAGA
1448 veri 5	CCGATTGAGAATGTTAAATCGATGC
1448 veri 3	CACGTCTATAAGCACCAATTGTG
pOS_iscC_hindIII 5	GGGAAGCTTGTCATGTTGTTTTCCTCCTAAGGATAC
pOS_isdC_kpnI 3	GCGGGTACCAACATAATCCTCCTTTTTATGATTGCTTTTA
pOS_fhuA_hindIII 5	GGGAAGCTTGGTATGAGCACACATACTTAAATAGAAGTCC
pOS_fhuA_kpnI 3	GCGGGTACCAATTTCCCTACTTTCAATAAAATTCTTTCTGT
HindIII tsr25p up	GGGAAGCTTCTTAAATATAGAATACTGTAACAC
kpnI tsr25p dwn	GCGGGTACCTTATACATTAGTGAGAATCATTGTC
Sbnpro3kpnI	GGGCCATGGGAATCTAACAATGAATCGTGACATGCTTGAC
Sbnpro5hindIII	GGGAAGCTTCCCACTACATCCTGCTAAAACAAGTAGG
ylaN5GSTBamHI	GGGGGATCCATGGCGAAACAAGCAACAATGAAAAATGCAG
5-yfp-ylaN	AAGCTAGCTCCGTCGACGCTTCAGAGATGATAGTTGACCATC
3-yfp-ylaN	GAATTAGCTTGCATGCTTATTTAGCTGTAAACGCGTCATG
ylaN3XhoI	GGGCTCGAGTTAAACAAGTGTAAAAGCTTCATGTAATTTTG
lacZ TIR up kpnI	CCCGGTACCATTTATAAGGAGGAAAAACATATGTCTA
lacZ TIR down EcoRI	TACGAATTCTTATTTTTGACACCAAACTAATTGATAA
fdh qPCR Fwd	GATGCGTTAGTTGAAGCGTTAG
fdh qPCR rev	ACCTGAATAGTGAACCGTCATAG
sbnB qPCR Fwd	GGACACATCGCAGATCGAATTA
sbnB qPCR rev	TCGATGGATTGTCGTGCTTAC
isdB qPCR Fwd	GACAATACCATGTCAGAATCGTTG
isdB qPCR rev	GCTGGAGTAGCTTCCTTCTTAG
UreA qPCR Fwd	AGCACGTGGTTTGAAACTAAATC
UreA qPCR rev	AACTCTGCAACGGTCTTACC
bioA qPCR Fwd	GTGATGAGGTAGCAGTTGGTT
bioA qPCR rev	GTGGTAAGTAGCCACCAGTAATC
nrdG qPCR Fwd	CAAAGCATTTCGAGCACGTT
nrdG qPCR rev	CTCTCGACGTTCTGTACAATCA
qoxD qPCR Fwd	TCCACGCGAAGTTGACAATTA
qoxD qPCR rev	AAACGTCCATCTTTACCTTCAGT
ddh qPCR Fwd	GCGTCGCTTCCCAGATATT
ddh qPCR rev	CGATACGACCCGTACCAATAAT
SAUSA300_0324 qPCR Fwd	CGCATACGTTAGGTGTCCAA
SAUSA300_0324 qPCR rev	CTATACCTAGTTCGGCAGCTATTT
SAUSA300_1658 qPCR Fwd	GGGTGAAACAAAGCCAGTTG
SAUSA300_1658 qPCR rev	AGGACGATTTGTGTCCATCC
SAUSA300_0986 qPCR Fwd	GCAACGATTGGTGTTGGTATG
SAUSA300_0986 qPCR rev	CAACTCCCACTGCTACAGTAAT
16srna FWD set3	TGAGTGCAGAAGAGGAAAGTG
16srna REV set3	CGTCAGTTACAGACCAGAAAGT
isdC FWD set 4	TCATCATCGCGACATTCAGTAA
isdC REV set 4	GCAATTGACGTGTCATTGGTATT
fhuD1 FWD set 4	TGGTCAAGCAACAGCATCT
fhuD1 REV set 4	GCATACCGAATGCATCATGAAC
Tsr25 qpcr fwd	ACGTTTCGTTCTTGTTGGATTG
Tsr25 qpcr rev	GTGTCGTAAGGGTTTACTGCT
sbnA FWD set 3	GAAAGATCGACCTGCCAAGTA
sbnA REV set 3	GCCAACGCAATGCCTAAAT
isdA FWD set 2	CAGAAGCTACGAACGCAACTA
isdA REV set 2	GACTTCTCTGAAGAGCCATCTTT
ylaN FWD set 2	GGCGAAACAAGCAACAATGA
ylaN REV set 2	TCTTCATATAATGGGCATGAAGGTA

isdC_2FurBox_EMSA_F	GATTAACCATTGCAATTGATAATGG
isdC_2FurBox_EMSA_R	CTTTAGTAAAATCATAAATAATAATG
BamHI_fur_ATG_FWD	GGATGCGGATCCATGGAAGAACGATTAAATCGCGTTAAGC
EcoRI_fur_REV	GCTCGCGAATTCCTATCCTTTACCTTTAGCTTGGCACG
IM515	TTTCCAATTCCTCCTCATCATACTCTATC
IM1360	GGTTCTAGTGGTGGTGGTGGTTCTGG
YlaN_pSmBIT_pLgBIT_F	GATAGAGTATGATGAGGAGGAATTGGAAAATGGCGAAACAAGCAACAATGAAAAATG
YlaN_pSmBIT_pLgBIT_R	GAACCACCACCACCACTAGAACCAACAAGTGTAAAAGCTTCATGTAATTTTG
Fur_pSmBIT_pLgBIT_F	GATAGAGTATGATGAGGAGGAATTGGAAAATGGAAGAACGATTAAATCGCGTTAAG
Fur_pSmBIT_pLgBIT_R	GAACCACCACCACCACTAGAACCTCCTTTACCTTTAGCTTGGCACGTTTC

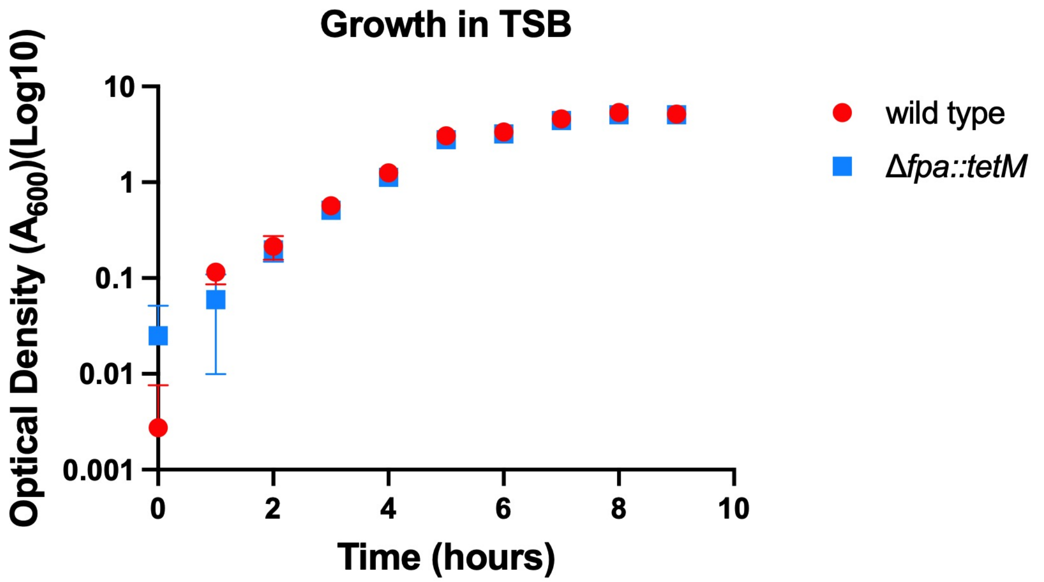

Figure S1. Growth of the wild type and Δ*fpa::tetM* strains in TSB medium. Overnight cultures of the wild type (JMB1100) (red circle) and the Δ*fpa::tetM* mutant (JMB8689) (blue square) were diluted 1:1000 into 5 mL of fresh TSB in 25 mL capacity culture tube. Cells were cultured at 37 °C with shaking at 200 rpm.

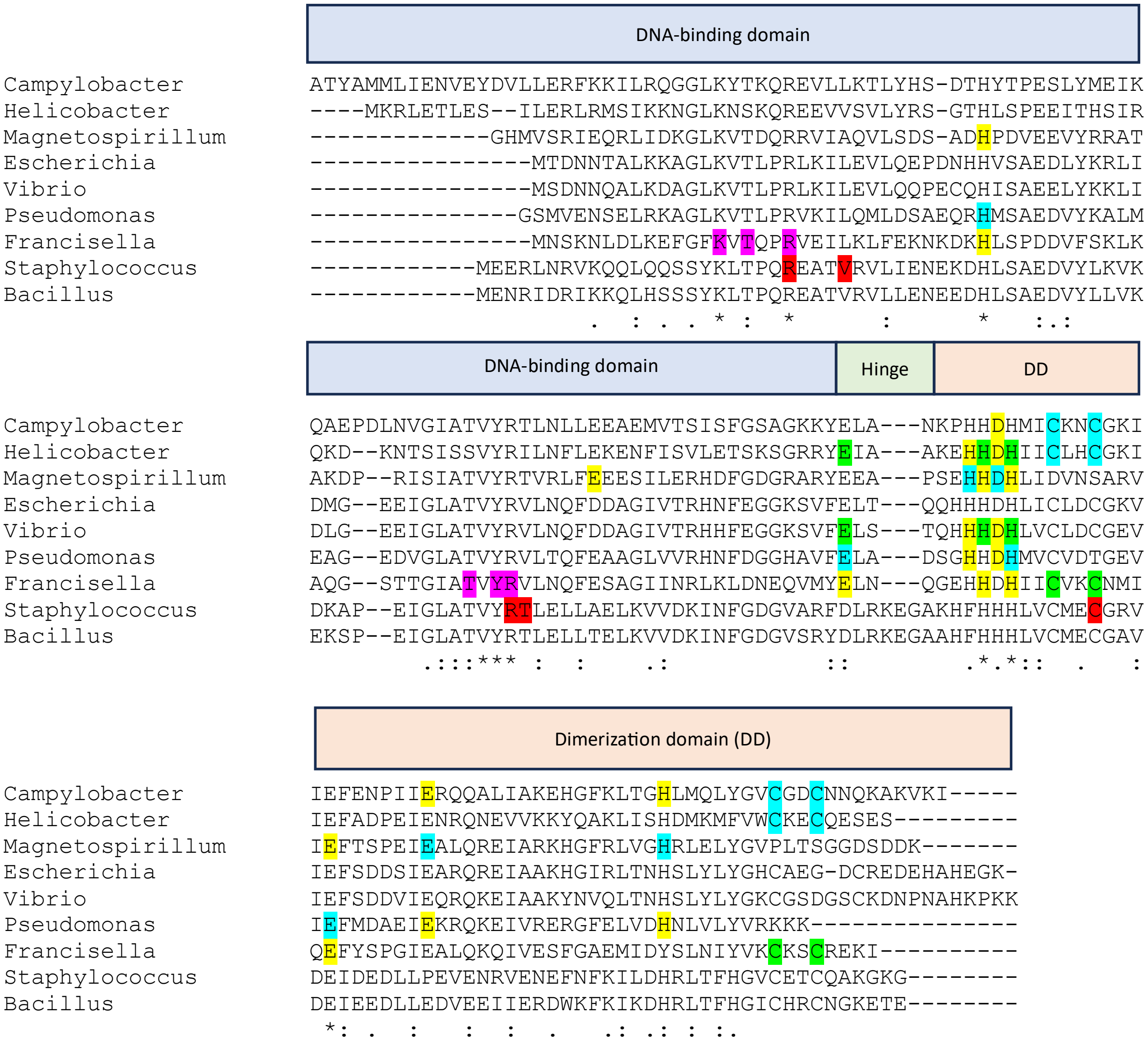

Figure S2. Alignment of Fur sequences from various bacteria. The amino acids that ligate the two Zn(II) ions in the *Campylobacter jejuni* Fur are highlighted in yellow and green (1). The amino acids ligate the three Zn(II) ions in the *Helicobacter pylori* Fur are highlighted in yellow, blue, and green (2). The amino acids that ligate the two Mn(II) ions in the *Magnetospirillum gryphiswaldense* Fur are highlighted in yellow and blue (3). The amino acids that ligate the two Zn(II) ions in the *Pseudomonas aeruginosa* Fur are highlighted in yellow and green (4). The amino acids that ligate the Fe(II) in the *Francisella tularensis* Fur is shown in yellow and the Zn(II) ligating amino acids are shown in green (5). The amino acids of the *F. tularensis* Fur that interact with DNA are highlighted in magenta. The sequences of the *B. subtilis*, *E. coli*, and *S. aureus* Fur are also included. The residues that are altered in the strains with second site mutation in *fur* that suppress the phenotypes of the *S. aureus* Δ*fpa* mutant are highlighted in red. Stars below the amino acid sequences indicate residues that are strictly conserved, and amino acids with dots underneath represent amino acids that are highly conserved. The DNA-binding, hinge, and dimerization domains of the *Magnetospirillum gryphiswaldense* Fur are highlighted.

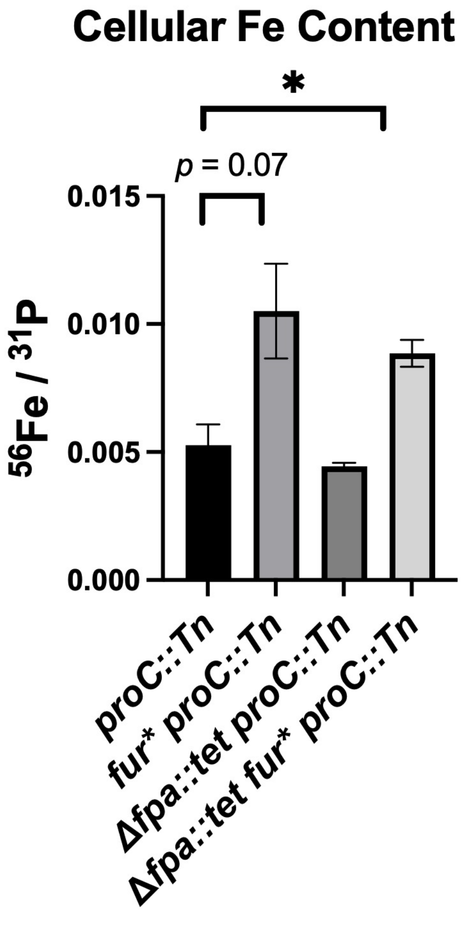

Figure S3. The ^{56}Fe and ^{31}P loads were quantified in whole cells using ICPMS after culture in TSB medium. The ratio of ^{56}Fe / ^{31}P is displayed for the *proC::Tn* (JMB10675), Δ*ylaN::tetM proC::Tn* (JMB10677), and *fur* proC::Tn* (JMB10676), and Δ*ylaN::tetM fur* proC::Tn* (JMB10678) strains are shown. The data displayed represent the average of biological triplicates with standard deviations shown. Student's t-tests were performed on the data and * indicates $p < 0.05$.

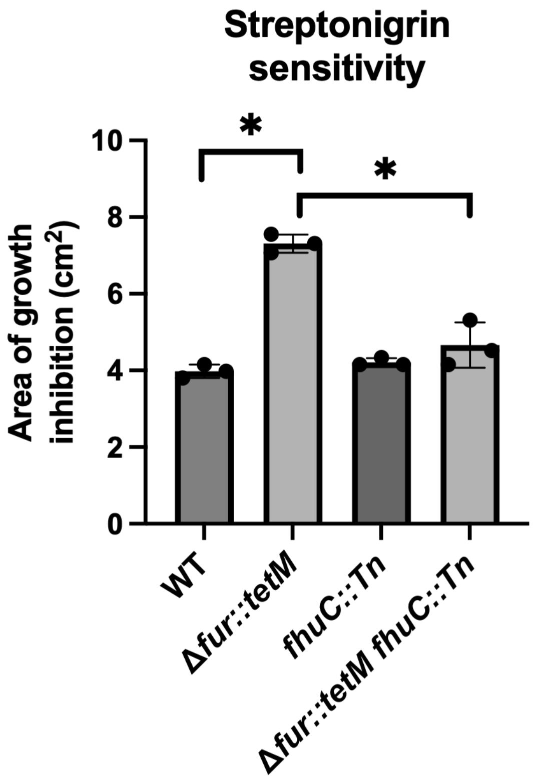

Figure S4. Introduction of a *fhuC::Tn* mutation decreases the streptonigrin sensitivity of the Δ*fur::tetM* strain. Streptonigrin sensitivity was monitored using top-agar tryptic soy agar overlays enclosing the WT (JMB1100), Δ*fur::tetM* (JMB1432), *fhuC::Tn* (JMB7525)*,* Δ*fur::tetM fhuC::Tn* (JMB10664) strains. Five µL of 2.5 mg mL^{-1} streptonigrin was spotted upon the overlays and the area of growth inhibition was measured after 18 hours of growth. The bars represent the average of three biological replicates with standard deviations displayed. Student's t-tests were performed on the data in panels C and D. * indicates $p < 0.05$.

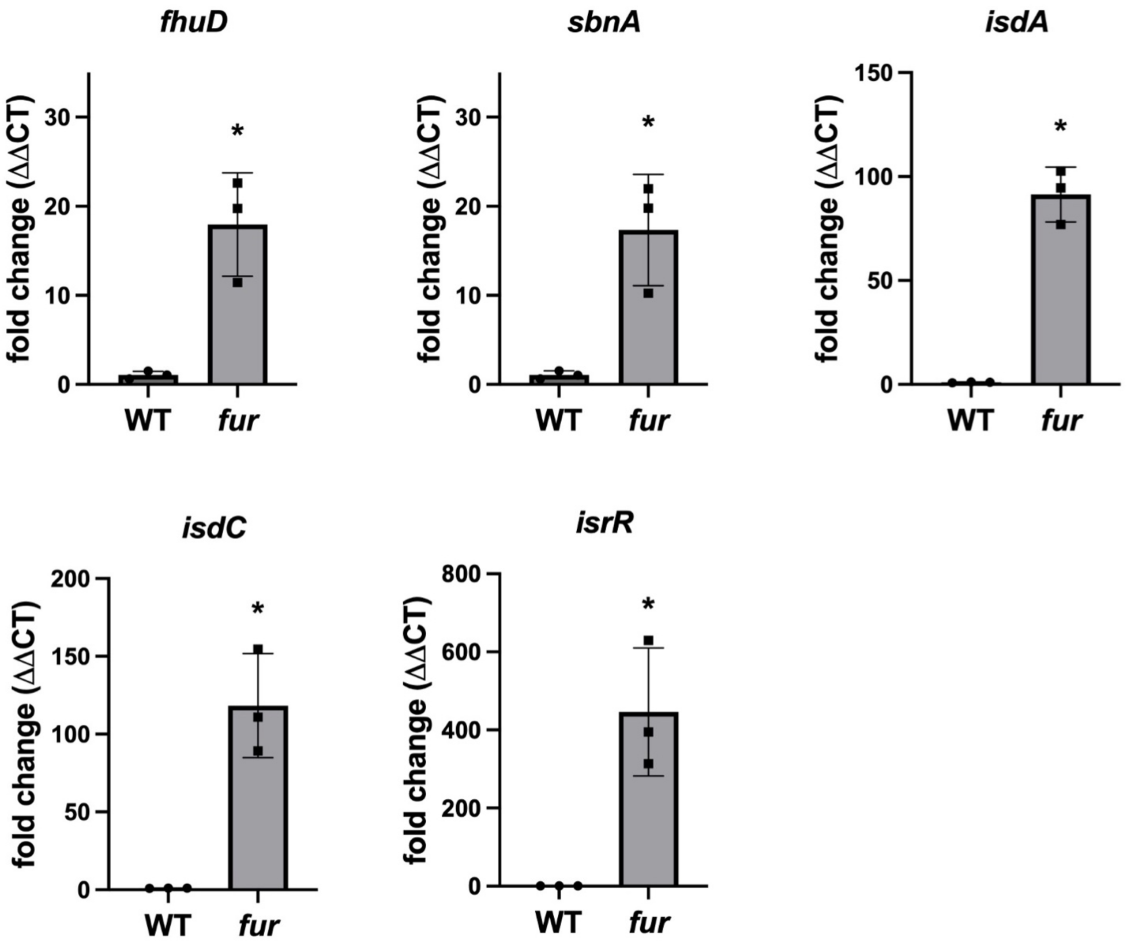

Figure S5. Verifying Fur regulated genes in *S. aureus*. The abundances of RNAs from the WT (JMB1100) and Δ*fur::tetM* (JMB1432) strains were determined using quantitative PCR. Fold changes were determined using the ΔΔCt method. The data displayed represent the average of biological triplicates with standard deviations shown. Student's t-tests were performed on the data and * indicates $p < 0.05$.

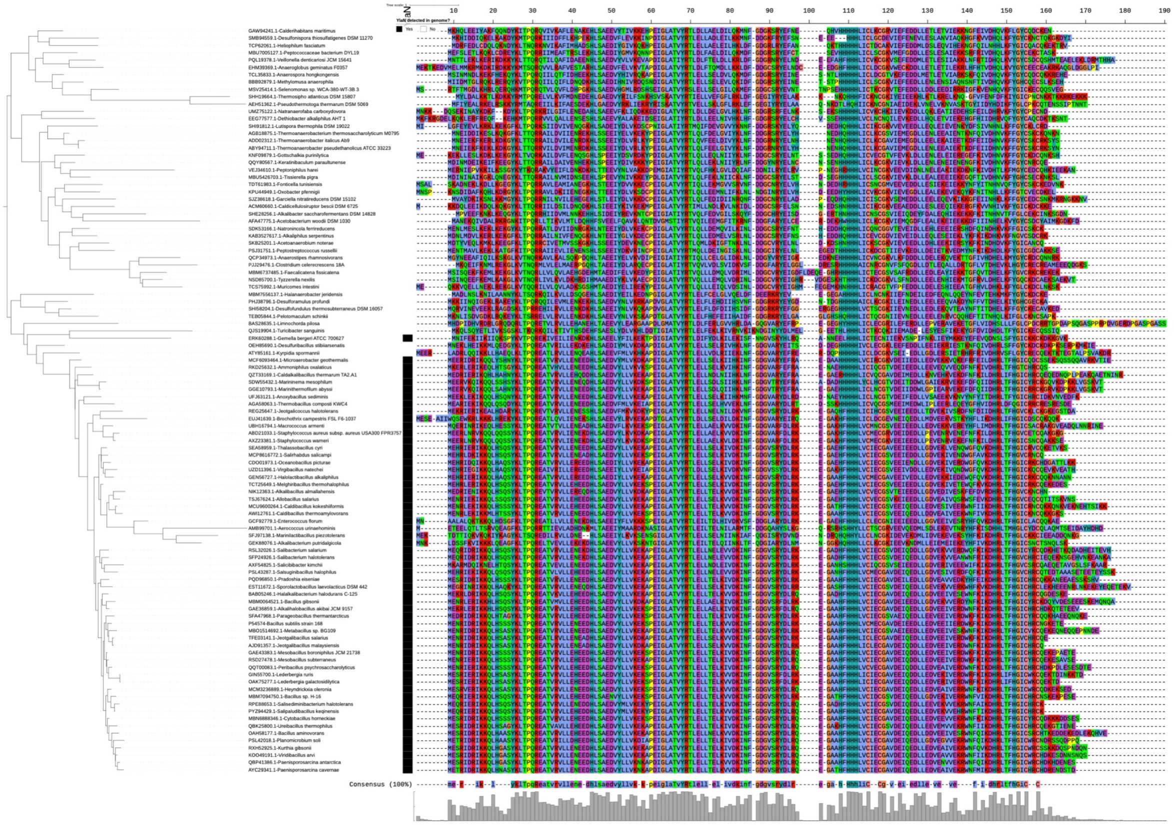

Figure S6: Down sampled phylogeny and associated alignment of Firmicutes Fur sequences. Sequences were randomly chosen from those in the Firmicutes Fur clade (Figure 4) to facilitate visualization of the relationship (tree shown on left side of the figure) between the YlaN-positive (black bars in the middle of the figure) and -negative sequences and their associated aligned protein sequences (left side of the figure; residues colored using the clustal scheme). Phylogeny were constructed using maximum-likelihood with automatic model selection and node support tested *via* 2,000 ultrafast phylogenetic bootstraps.

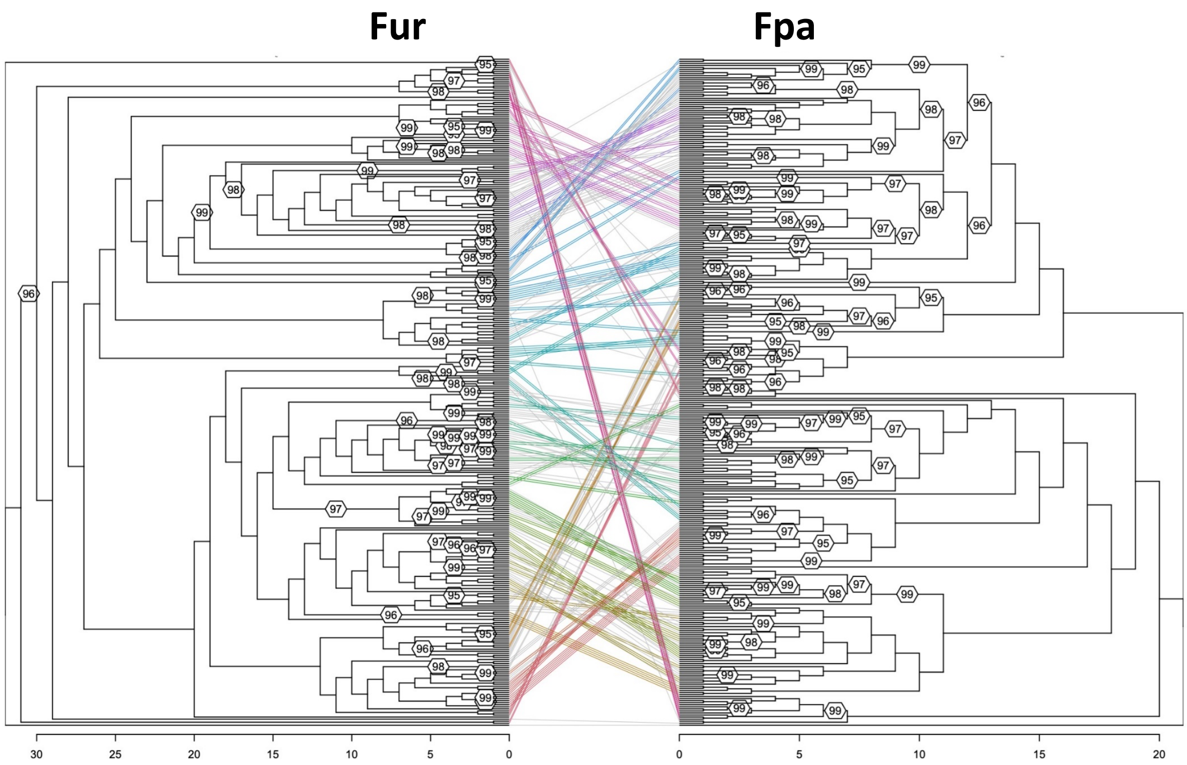

Figure S7: Tanglegram of Firmicutes Fur and Fpa proteins. Phylogenies of Firmicutes Fur (left) and Fpa (right) proteins from Fpa-positive genomes. Lines are used to connect the same tips in each tree, with groups of tips from clades shared between both trees shown using colored lines. Phylogenies were constructed using maximum-likelihood with automatic model selection and node support tested *via* 2,000 ultrafast phylogenetic bootstraps. Branch lengths have been scaled and only nodes with ≥ 95% bootstrap support are annotated to improve readability.

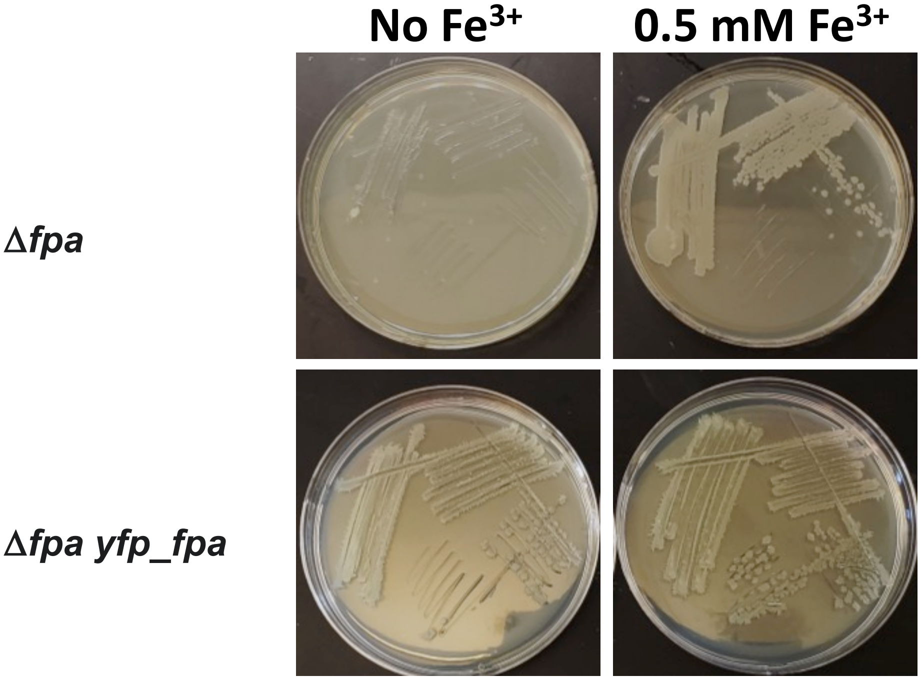

Figure S8. The Yfp-Fpa chimeric protein is functional *in vivo*. The plasmid p*Mutin4_fpa* was used to transform wild type (BD630) and a strain harboring *yfp_fpa* (BD8592). This plasmid will integrate into the *Bacillus* chromosome by Campbell integration at the native *fpa* gene, putting the downstream genes under the control of the IPTG-inducible promoter. *fpa* is essential in *B. subtilis*, but *fpa* mutants can grow in the presence excess iron salts, bypassing its essentiality. Following transformations, cells were plated on LB plates with appropriate antibiotics, 0.5 mM IPTG and 0.5 mM $FeCl_3$. The resulting transformants, Δ*fpa yfp_fpa* (VCB102) and Δ*fpa* (VCB103), were purified on plates with or without excess iron. Pictures of a representative experiment are displayed.

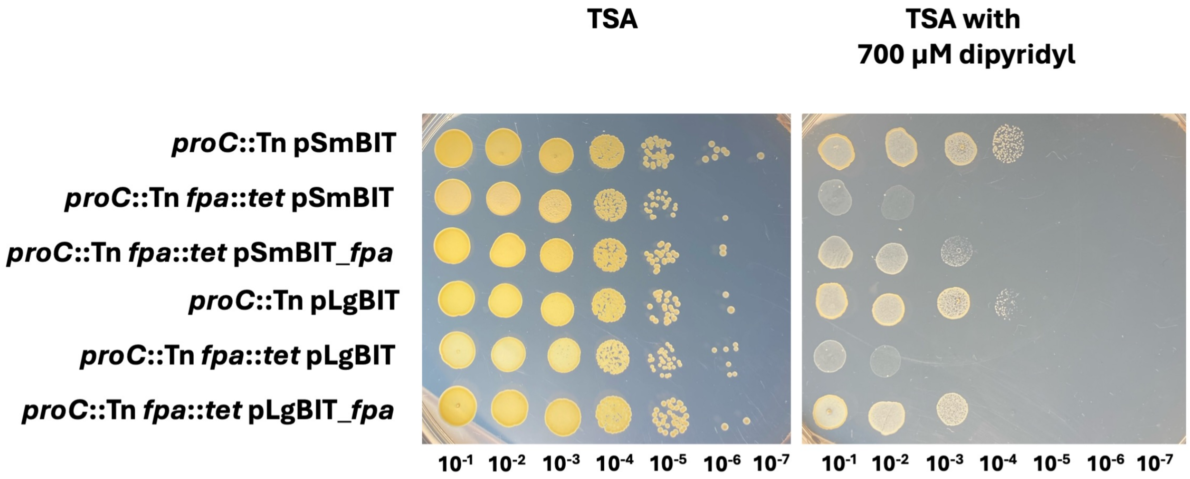

Figure S9. The SmBIT-Fpa and LgBIT-Fpa chimeric proteins are functional *in vivo*. Growth of the *proC::Tn* (JMB10675) or *proC*::Tn Δ*fpa::tetM* (JMB10677) strains containing pSmBIT, pSmBIT_*fpa*, pLgBIT, or pLgBIT_*fpa* on solid TSB with or without 700 μM 2,2-dipyridyl (DIP). Overnight cultures were grown in TSB with chloramphenicol or kanamycin, serially diluted, and spot-plated. Pictures of a representative experiment are displayed.

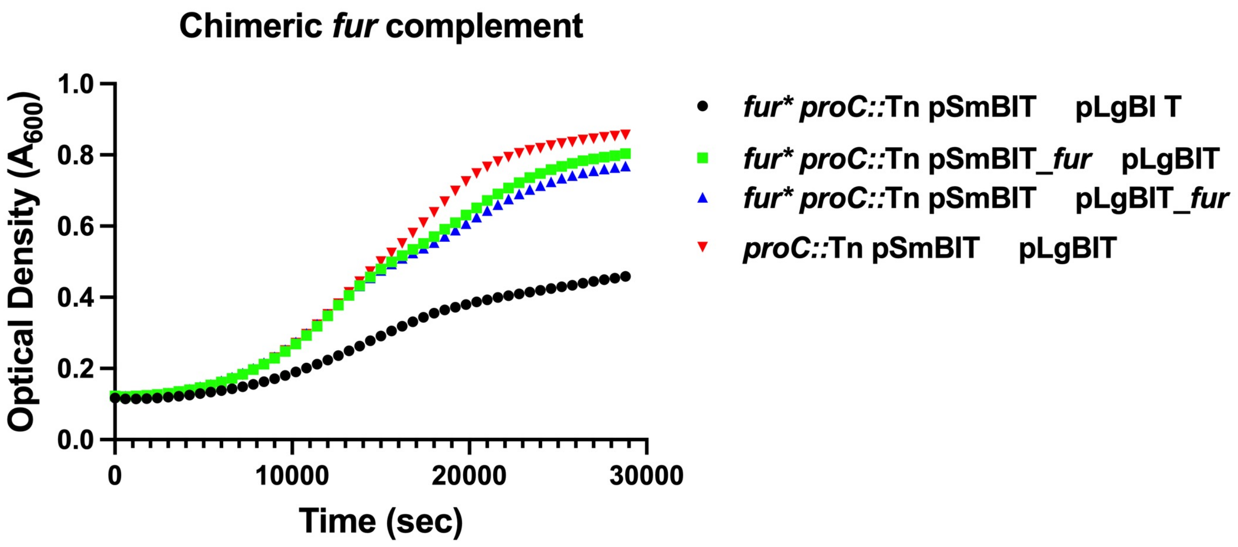

Figure S10. The SmBIT-Fur and LgBIT-Fur chimeric proteins are functional *in vivo*. The *proC::*Tn (JMB10675) or *proC::*Tn *fur* fpa::tetM* (JMB10678) strains containing a combination of pSmBIT, pSmBIT_*fur*, pLgBIT, and pLgBIT_*fur* were cultured in TSB medium containing chloramphenicol, kanamycin, and anhydrotetracycline (5 ng μL^{-1}) to induce expression. Cultures contained 200 μL of medium in a 96-well plate. Cell density was monitored at 600 nm over time. The data represent the average of biological triplicates with standard deviations displayed. The error bars are smaller than the data points.

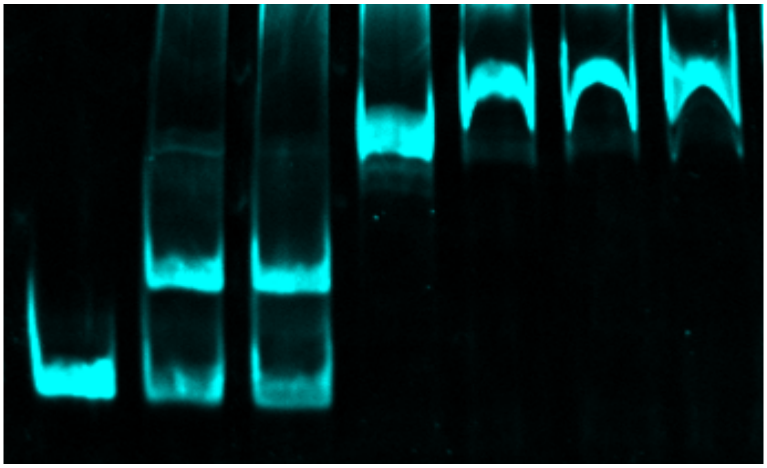

Figure S11. **Fur binds the *isdC* operator.** Titration of Fur (0 – 100 µM) in an electrophoretic mobility shift assay (EMSA) binding assay with the *isdC* operator DNA which contains two consensus Fur box sequences. A photograph of a representative experiment is displayed.

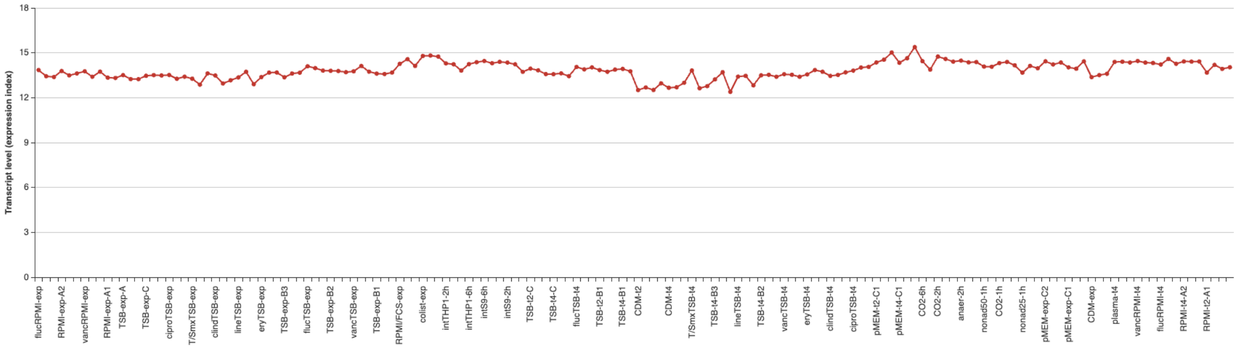

Figure S12. *fpa* transcript accumulation under a variety of growth conditions. Transcript abundances corresponding to *fpa* were quantified in *S. aureus* strain HG001 after culture in a variety of media and growth conditions. The data represent the average of biological triplicates. These data were originally published by Mäder et al. (6) and the figure was generated from AureoWiki website (7).

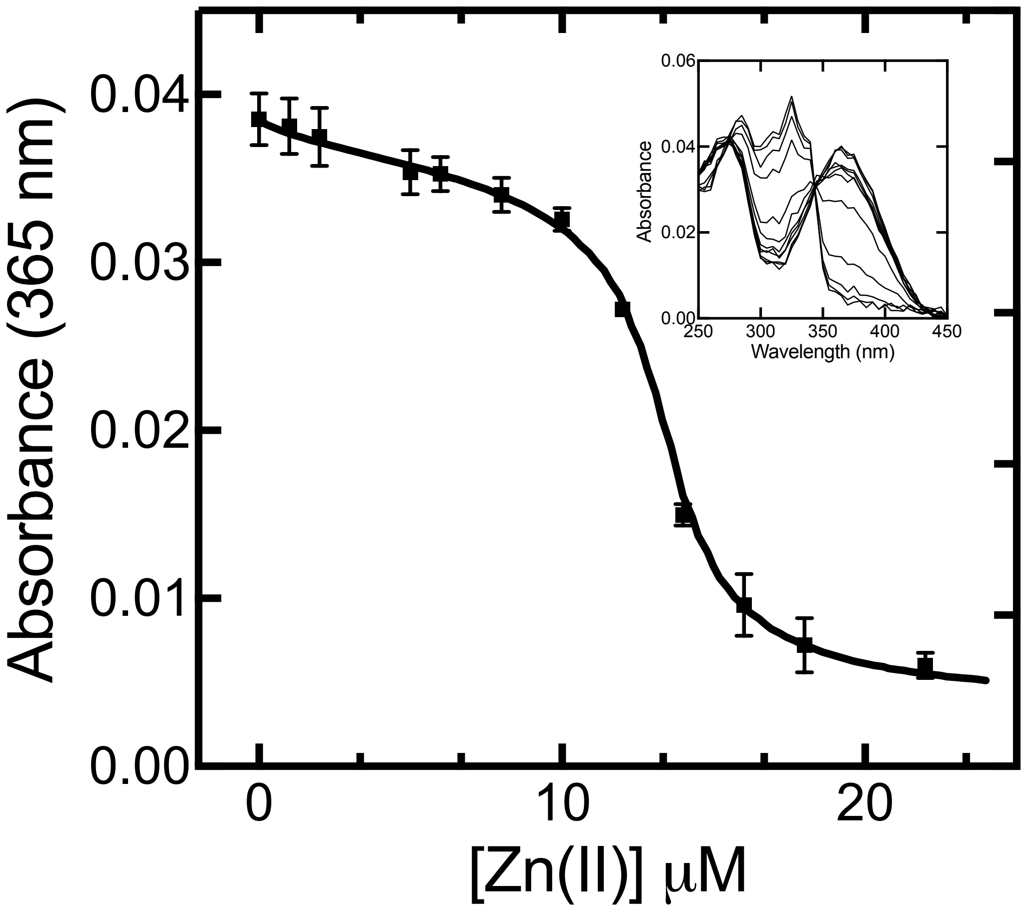

Figure S13. Zn(II) binding affinity to Fpa measured using Mag-Fura-2 within a competition-based assay. Zn(II) binding affinity to Fpa measured using Mag-Fura-2 within a competition-based assay. Average titration spectral points of Zn(II) ions into Fpa with overall simulation in solid line with representative titration absorption spectra inset display. Mag-fura-2 to protein ratios were varied and 1:1 data is shown. Spectra were collected in triplicate using independent samples to ensure spectral reproducibility.

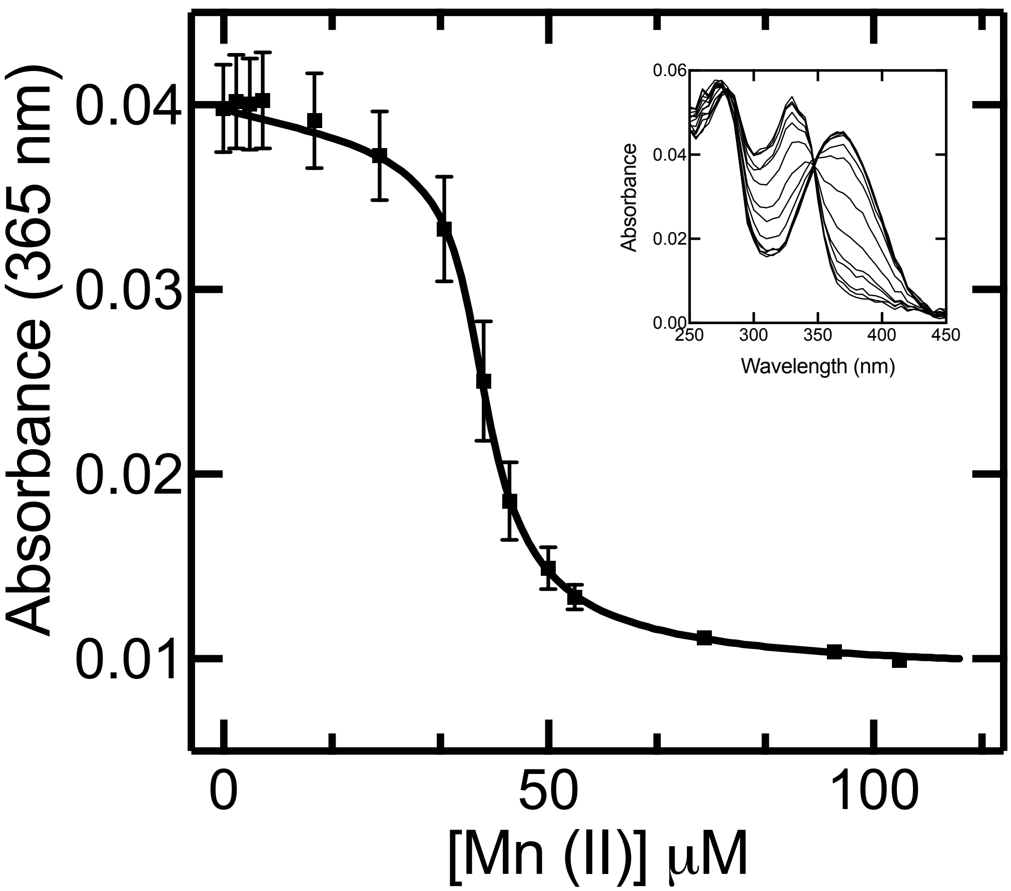

Figure S14. Mn(II) binding affinity to Fpa measured using Mag-Fura-2 within a competition-based assay. Mn(II) binding affinity to Fpa measured using Mag-Fura-2 within a competition-based assay. Average titration spectral points of Mn(II) ions into Fpa with overall simulation in solid line with representative titration absorption spectra inset display. Mag-fura-2 to protein ratios were varied and 1:1 data is shown. Spectra were collected in triplicate using independent samples to ensure spectral reproducibility.

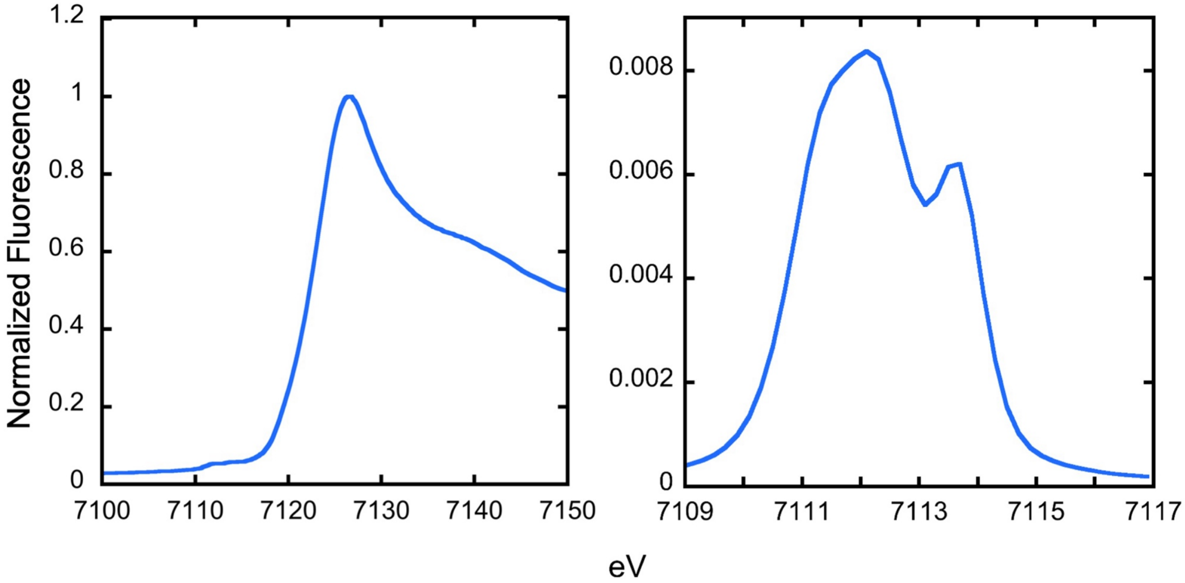

Figure S15. **Examining the Fpa Fe(II) ligand environment.** Normalized XANES for Fe(II) bound to Fpa. Representative spectra for Fe XANES for Fe-Fpa (left) and extended display of Fe-XANES pre-edge region (right).

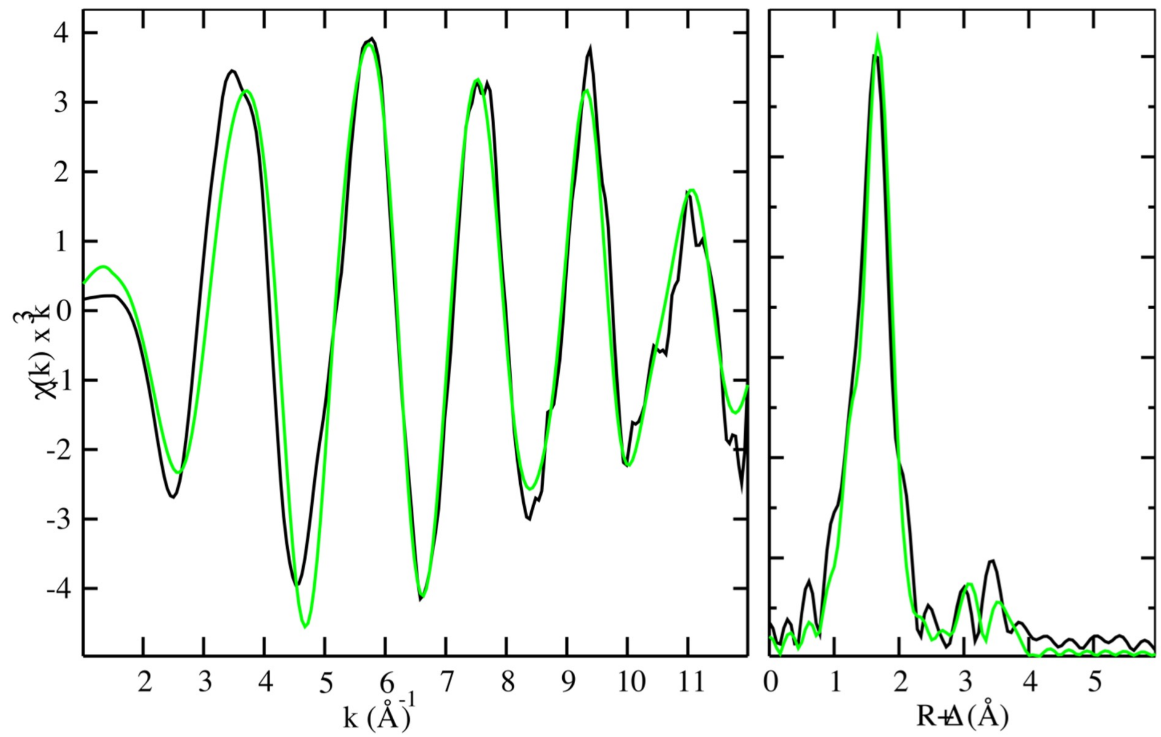

Figure S16. **Examining the Fpa Fe(II) ligand environment.** EXAFS analyses of Fe(II) bound Fpa. Raw EXAFS, Fourier transforms of EXAFS, and spectral simulations for Fe(II) bound Fpa. Fe EXAFS data and simulation of Fpa. Raw k^3 weighted EXAFS data (left) and phase-shifted Fourier transform (FT) (right) are shown. Raw data is shown in black, and simulations in green.

Works Cited:

1. Butcher J, Sarvan S, Brunzelle JS, Couture JF, Stintzi A. 2012. Structure and regulon of *Campylobacter jejuni* ferric uptake regulator Fur define apo-Fur regulation. Proc Natl Acad Sci U S A 109:10047-52.
2. Dian C, Vitale S, Leonard GA, Bahlawane C, Fauquant C, Leduc D, Muller C, de Reuse H, Michaud-Soret I, Terradot L. 2011. The structure of the *Helicobacter pylori* ferric uptake regulator Fur reveals three functional metal binding sites. Mol Microbiol 79:1260-75.
3. Deng Z, Wang Q, Liu Z, Zhang M, Machado AC, Chiu TP, Feng C, Zhang Q, Yu L, Qi L, Zheng J, Wang X, Huo X, Qi X, Li X, Wu W, Rohs R, Li Y, Chen Z. 2015. Mechanistic insights into metal ion activation and operator recognition by the ferric uptake regulator. Nat Commun 6:7642.
4. Pohl E, Haller JC, Mijovilovich A, Meyer-Klaucke W, Garman E, Vasil ML. 2003. Architecture of a protein central to iron homeostasis: crystal structure and spectroscopic analysis of the ferric uptake regulator. Mol Microbiol 47:903-15.
5. Perard J, Nader S, Levert M, Arnaud L, Carpentier P, Siebert C, Blanquet F, Cavazza C, Renesto P, Schneider D, Maurin M, Coves J, Crouzy S, Michaud-Soret I. 2018. Structural and functional studies of the metalloregulator Fur identify a promoter-binding mechanism and its role in *Francisella tularensis* virulence. Commun Biol 1:93.
6. Mader U, Nicolas P, Depke M, Pane-Farre J, Debarbouille M, van der Kooi-Pol MM, Guerin C, Derozier S, Hiron A, Jarmer H, Leduc A, Michalik S, Reilman E, Schaffer M, Schmidt F, Bessieres P, Noirot P, Hecker M, Msadek T, Volker U, van Dijl JM. 2016. *Staphylococcus aureus* Transcriptome Architecture: From Laboratory to Infection-Mimicking Conditions. PLoS Genet 12:e1005962.
7. Fuchs S, Mehlan H, Bernhardt J, Hennig A, Michalik S, Surmann K, Pane-Farre J, Giese A, Weiss S, Backert L, Herbig A, Nieselt K, Hecker M, Volker U, Mader U. 2018. AureoWiki-The repository of the *Staphylococcus aureus* research and annotation community. Int J Med Microbiol 308:558-568.
